# Motherhood and mental health of adolescent girls in low- and middle-income countries: A scoping review

**DOI:** 10.1371/journal.pgph.0005134

**Published:** 2025-09-17

**Authors:** Amber Hussain, Yared Asmare Aynalem, Tanya Park, Salima Meherali

**Affiliations:** 1 Faculty of Nursing, University of Alberta, Edmonton, Canada; 2 College of Health Science, Debre Berhan University, Debre Berhan, Ethiopia; 3 College of Healthcare Sciences, Nursing and Midwifery, James Cook University, Australia; Dalhousie University, CANADA

## Abstract

Adolescence, a crucial transitional period, involves significant physical, cognitive, and psychosocial transformations. In Low- and Middle-Income Countries (LMICs), adolescent pregnancy is a significant public health concern, with an estimated 95% of the world’s adolescent births occurring in these settings. While extensive research has explored various facets of adolescent development, there remains a gap in understanding mental health challenges experienced during motherhood. This scoping review aimed to assess the existing literature on the mental health of adolescent mothers in LMICs and identify gaps to guide future research and interventions in this underexplored domain. A scoping review was conducted following the methodology outlined by Arksey and O’Malley methodology. Relevant studies were retrieved from electronic databases (CINAHL, EMBASE, MEDLINE, Global Health, ERIC, and PsycINFO) and grey literature sources, using search terms systematically mapped with the PCC (Population, Context, and Concept) format. The population (P) includes adolescents aged 10–19 years; Context (C) includes Low- and Middle-Income Countries; and Concept (C) includes motherhood and mental health. A two-stage screening process was employed using Covidence software, with conflicts resolved through consensus or consultation with a third reviewer. Data extraction was performed by the primary author and independently reviewed by a second author. The findings were analyzed using descriptive statistics and narrative description. 1240 articles were identified, of which 35 studies met the inclusion criteria. Most studies focused on the postnatal phase, with limited attention to antenatal and childbirth. Mental health issues were the primary focus (88.6%), while some explored factors influencing mental health (57.1%), healthcare access and utilization (8.6%), interventions (2.9%), and coping strategies (8.6%). A majority of studies lacked a specified theoretical framework (85.7%). Most of the studies were from Sub-Saharan Africa. This review provides valuable insights for future research, policy development, and interventions addressing the mental health needs of adolescent mothers in LMICs. It highlights the need for adolescent-responsive mental health policies, integration of mental health services into maternal care, and culturally tailored interventions such as community-based peer support and mobile health tools. Future research should adopt theory-informed, context-specific approaches and expand into underrepresented regions, using qualitative and longitudinal designs to examine the full continuum of motherhood encompassing antenatal, childbirth, and postnatal phases.

## Introduction

Adolescence (ages 10–19) is a critical stage of development, marked by physical, cognitive, and social transitions that impact mental well-being [1]. While adolescent motherhood is recognized as a global public health concern, its mental health implications remain underexplored, particularly in Low- and Middle-Income Countries (LMICs), where socioeconomic vulnerabilities, health system limitations, and cultural norms exacerbate psychological stress [2].

Motherhood extends beyond fertility, encompassing pphysical, emotional, and social responsibilities during pregnancy, childbirth, and postpartum period [3–8]. The World Health Organization (WHO) defines safe motherhood as ensuring all women receive the care they need to remain safe and healthy throughout pregnancy and childbirth, including antenatal, intrapartum, postnatal, newborn, and mental health care [9]. For adolescent mothers, this includes not only physical complications but also unique psychosocial challenges arising from their dual developmental transitions.

Over 95% of global adolescent births occur in LMICs [10]. The burden of adolescent pregnancies are disproportionately high in LMICs, driven by poverty, gender inequality, early marriage and limited access to education and healthcare [2,10–16], all of which reflect broader violations of adolescents’ human rights, including their rights to autonomy, safety, and health. These systemic barriers often force young girls into adult roles prematurely without adequate emotional readiness or social support. Compared to older mothers, adolescents are more vulnerable to depression, anxiety, and post-traumatic stress disorders, which, in turn, can negatively impact child health and development [17–21].

Existing literature on adolescent motherhood in LMICs tends to focus on physical and reproductive health outcomes, with less emphasis on psychological well-being. This scoping review is therefore necessary to determine what is currently known about the mental health of adolescent mothers during the motherhood transition in LMICs, and to highlight gaps in culturally relevant research and service provision [22,23]. The objective of this scoping review is to synthesize existing evidence on the mental health of adolescent mothers during the motherhood transition in LMICs. By consolidating current knowledge, we can identify existing gaps in understanding this important developmental period. Mapping the literature will provide insights into mental health challenges, guide future research, and identify strategies for improving adolescent mothers’ mental well-being.

The review addresses the following questions developed using the PCC (Population, Concept, Context) framework: (1) What is known from the existing literature about the mental health of adolescent mothers during the motherhood transition in LMICs? (2) To what extent does literature explore the mental health of adolescent mothers during the motherhood transition in LMICs (3) What are the gaps in the existing literature about the mental health of adolescent mothers during the motherhood transition in LMICs?

## Methods

### Study design

A scoping review was conducted following the methodology outlined by Arksey and O’Malley (2005) [24], and advanced by Levac et al., (2010) [25]. This framework involves the following steps: (1) formulating a research question, (2) identifying pertinent studies, (3) selecting studies, (4) organizing data, (5) compiling, summarizing, and presenting the findings, and (6) an optional consultation exercise. We did not incorporate the optional step of expert consultation due to time and financial constraints. The selection of a scoping review methodology stems from the fact that the mental health of adolescent mothers had not undergone a comprehensive review within the context of LMICs before. This approach is particularly valuable for exploring broadly covered subjects, facilitating a thorough and systematic mapping of the literature, and identifying key concepts, evidence, or research gaps in the specific area of interest [24]. To ensure transparency and reproducibility, we followed the Preferred Reporting Items for Systematic Reviews and Meta-Analyses Extension for Scoping Reviews (PRISMA-ScR) guidelines for reporting this review. The review protocol was registered on the Open Science Framework on August 31, 2023, at https://doi.org/10.17605/OSF.IO/BZQSW.

### Data sources and search strategy

A comprehensive search strategy was developed in consultation with a Health Sciences Librarian (MK). Relevant studies were retrieved by searching electronic databases, including CINAHL, EMBASE, MEDLINE, Global Health, ERIC, and PsycINFO. In addition, a targeted grey literature search was conducted, including ProQuest Dissertations & Theses, to capture unpublished theses and dissertations related to adolescent motherhood and mental health (refer to S1 File). The reference lists of all selected articles for full-text review were meticulously screened for additional papers, augmenting the search strategy’s coverage [25]. In collaboration with the librarian, search key terms were systematically mapped using the PCC (Population, Context, and Concept) format, facilitating the identification of pertinent articles across four primary categories: 1) Low- and Middle-Income Countries, 2) adolescent, 3) motherhood, and 4) mental health, utilizing a combination of synonymous and free-text terms. Medical Subject Headings (MeSH) terms were retrieved for the important concepts, including “mental health” and “motherhood.” The search, inclusive of a variety of terms, was not restricted by publication year to ensure a comprehensive exploration of literature and minimize potential bias in article selection. However, the search strategy was limited to studies available in English due to time, cost, and resource constraints. A comprehensive literature search was initially conducted between August 6–12, 2023, and updated on July 31, 2024, to ensure the inclusion of the most recent studies. No studies published in 2024 met the inclusion criteria at the time of review.

### Eligibility criteria

The targeted population comprised adolescent women aged 10–19 years. Studies with a broader age range were included if they provided data separately for adolescents. In terms of concept, articles were considered if they explored the mental health of adolescents during the motherhood transition, encapsulating two central concepts: (1) mental health, and (2) motherhood. Motherhood included any stage of the transition, such as antenatal, birth, and postnatal periods. The concept of mental health included but not limited to mental health symptoms, psychological well-being, and mental illnesses. The context was LMICs as defined by the World Bank (2020) based on Gross National Income (GNI) per capita [26]. The review included original research employing quantitative, qualitative, and mixed-method designs, as well as grey literature. Non-primary research was excluded, such as opinions, editorials, and commentaries to ensure that the review remained grounded in empirically derived evidence. Studies not published in English were also excluded due to resource constraints.

### Study selection

Two authors (AM and YA) independently employed a two-stage screening process for all records using Covidence software [27]. Prior to screening, duplicate records were automatically identified and removed using Covidence. The screening involved an initial review of titles/abstracts followed by a subsequent screening of full texts to determine inclusion. Conflicts were resolved by consensus between both reviewers. Unresolved disagreements were addressed through consultations with the third reviewer (TP), who has clinical and academic expertise in mental health nursing, women’s health, and adolescent well-being.

### Data extraction

The data extraction tool was adopted from the Joanna Briggs Institute (JBI) methodology guidance for scoping reviews [28]. To ensure rigor and clarity, several measures were undertaken. These included review of the extraction form by the research team, iterative refinement during the early stages of data extraction, and validation of extracted data by a second reviewer. The primary author (AH) extracted the data, and another author (YA) subsequently validated the charted data for accuracy and completeness. The information extracted included authors, year of publication, country and setting, study aim, theoretical framework used, methodological elements (study design, data collection method, target population, sample size, and age), and findings (refer to Table 1). Quality appraisal was not conducted since it is not required in scoping reviews according to the guidance provided on scoping review methodology [25,28,29]. The aim of this review was to identify and describe the nature of studies on the mental health of adolescent mothers, rather than to evaluate their quality.

**Table 1:** Publication details of all articles included in the scoping review after full-text screening.

Authors and Year	Country, Region and Income Level	Aim of study	Theoretical Framework	Study Design, Method, sampling techniques	Target Population, Age & Sample size	Main Findings
Agampodi et al., 2021	Sri Lanka, Rural (Anuradhapura) Income level: Lower middle income Region: South Asia	To describe the hidden burden, associated biological and psychosocial factors and utilization patterns of pre-conceptional services among pregnant adolescents in rural Sri Lanka.	None	Quantitative prospective cohort Study, self-administered questionnaire (Edinburgh Postpartum Depression Scale) Sampling technique not mentioned	Pregnant adolescent women (25–28 weeks of gestation), 15–19 years n = 233	Adolescent mothers were less happy of being pregnant (p = 0.006) and had significantly higher levels of anxiety (p = 0.009). Depression (p = 0.461) and anhedonia (p = 0.416)
Ajayi et al., 2023	Burkina Faso and Malawi Urban and rural areas Income level: Both Low income Region: Sub-Saharan Africa	To examine the socio-ecological factors associated with depression symptoms among pregnant and parenting adolescent girls.	Socioecological model	Quantitative Cross-sectional Global Early Adolescent Study tools, and Patient Health Questionnaire-9 (PHQ-9) Two-stage probability sampling (Random and household listing)	Pregnant and parenting girls, 10-19 years n = 669	Probable depression rates were 18.8% in Burkina Faso and 14.5% in Malawi. In Malawi, secondary education correlated with lower depression likelihood (AOR: 0.47), while in Burkina Faso, it didn’t. Family factors, such as denying paternity and lack of parental support, increased depression odds.
Belete et al., 2021	Ethiopia Public Hospital Income level: Low income Region: Sub-Saharan Africa	To assess the prevalence and factors associated with suicide ideation and attempt among pregnant women attending antenatal care services at public hospitals in southern Ethiopia.	None	Quantitative Cross-sectional Composite International Diagnostic Interview (CIDI) by WHO, SRQ-20 by WHO, and WHO violence measure Systematic random sampling	Pregnant women, 15-49 years Total participants = 762 Adolescent sample (15–19 years) n = 28	For 15–19 years:4 (14.3) had current suicidal ideation, COR = 1.78 (0.53-5.98), AOR = 1.23 (0.25-6.03)
Kassa et al, 2021	Ethiopia Seven districts in the East Gojjam zone, Northwest. Income level: Low income Region: Sub-Saharan Africa	To assess the adverse maternal outcomes of adolescent pregnancy in Northwest Ethiopia	None	Quantitative, Prospective Cohort Edinburgh Postnatal Depression Scale (EPDS) Multistage sampling	Adolescent and adult women adolescents’ age 15-19 years Total participants = 1,134 Adolescent sample (15–19 years): n = 374	A considerably larger proportion of teenage women experienced PPD (37.4%) compared to adult women (20.1%), p-value <0.000
Kaye, 2008	Kampala, Uganda Mulago hospital Income level: Low income Region: Sub-Saharan Africa	explore what adolescents perceived as their struggles during the period of transition from childhood to parenthood and specifically, describe strategies employed in coping with stress of pregnancy, motherhood, and parenthood.	Stress and coping model	Qualitative Longitudinal study In-depth interviews and focus group discussions. Theoretical sampling	Pregnant adolescents followed from pregnancy to delivery, 14–19 years. n = 52	Adolescents reported anxiety, loss of self-esteem, difficulty in accessing financial, moral, and material support from parents or partners and stigmatization by health workers when they sought care from health facilities. Three strategies by which adolescent mothers cope with parenting and pregnancy stress that were described as utilizing opportunities, accommodating the challenges, or failure, and varied to the extent to which they enabled adolescents to cope with the stress.
Khanna, 2021	India Rural Income level: Lower middle income Region: South Asia	To determine whether gender disadvantage factors are associated with psychological distress among young women in rural India.	None	Quantitative Cross-sectional GHQ-12 item questionnaire. Systematic random sampling	Young married women, 15–24 years n = 229	Psychological distress was found among 21.9%. Young women who were married before 18 years had 2.19 times higher odds of distress than women who were married after 18 years. Young women who gave birth to a female infant had 2.43 times higher odds of distress than those who gave birth to a male infant. Lack of partner support and experience of postnatal health complications were other predictors.
Kimbui et al., 2018	Kenya Nairobi, Health Centre Income level: Lower middle income Region: Sub-Saharan Africa	To identify social determinants of mental health such as social support, partner or parent support, and demographic profile	None	Quantitative cross-sectional Edinburgh Postnatal Depression Screen questionnaire Purposive Sampling	Pregnant adolescents, 16-18 years n = 212	43.1% had depression, among which 60.4% had depressive symptoms, and 51.9% had severe depression scores. 26.9% were currently consuming alcohol. The more severely depressed participants had greater alcohol use. Of the 110 pregnant adolescents who were severely depressed, 39 were currently consuming alcohol. Alcohol use factors associated with depression included living with an alcoholic, ever and current use of alcohol, alcohol-related harm being experienced, being pressured to take alcohol
Klingberg-Allvin et al., 2008	Vietnam Rural district Income level: Lower middle income Region: East Asia & Pacific	To explore adolescents’ perceptions and experiences related to transition into motherhood and their encounter with health care service.	None	Qualitative In-depth semi structured interviews The sampling technique not mentioned	Pregnant or newly delivered adolescent, less than 20 years n = 22	young women experienced ambivalence in the transition to motherhood in that they felt too young but also happy to be able to please their husband and the extended family. However, participants experienced lacking power with regard to decisions in relation to pregnancy, delivery, and contraceptive usage. They also had feelings of being patronized and ignored in the encounter with health care providers.
Kola et al., 2020	Nigeria Income level: Lower middle income Region: Sub-Saharan Africa	To identify factors influencing health service utilization for adolescent perinatal depression, in Nigeria to inform new strategies of care delivery.	Behavioral Model	Qualitative Focus group. Purposive sampling	Adolescent mothers with perinatal depression, age not mentioned. Also included providers n = 17 mothers and 25 providers	Perceived health benefits of treatment received for perinatal depression were strong motivation for service use. The service environment was often discouraging, with long queues and stigmatizing attitude by many of the providers. In the clinic, the stigma was about both their early pregnancy and their depression.
Labrague et al., 2020	Philippines Rural area Income level: Lower middle income Region: East Asia & Pacific	To explore the prevalence and predictors of postpartum depression (PPD) as well as the utilization and evaluation of PPD services among postpartum women in rural areas of the Philippines.	None	Quantitative Cross-sectional study Edinburgh Postnatal Depression Scale (EPDS) Convenience Sampling	Women who visited maternal facilities (no age limit) Total sample = 165, Adolescent less than 19 years n = 39	Age < 19 years (N = 39): 9 (23.1%) had PPD
Li et al., 2021	Bangladesh Urban & Rural areas Income level: Lower middle income Region: South Asia	To assess the prevalence of suicide attempts among young women with adolescent pregnancy in Bangladesh and to explore its associated factors	None	Quantitative Cross-sectional study Suicide-Screening Questions Toolkit Sampling not mentioned	Pregnant women, Less than 18 years n = 940	6.5% reported suicide attempts in the past 12 months, and the majority (88.5%) of the attempts happened within one year after the pregnancy. Participants with more years after first pregnancy and more perceived social support from friends were less likely to have suicide attempts, and those perceived bad health status compared with good/fair health status were more likely to attempt suicide.
Mark et al., 2021	Malawi Rural Ntcheu District Income level: Low income Region: Sub-Saharan Africa	To evaluate the association between food insecurity (FI) and clinical depression and the modifying effects of seasonality on this association	None	Quantitative, Cross-sectional Food insecurity: USAIDs Household Food Insecurity Access Scale (HFIAS). PPD: Chichewa version of the Self-Reporting Questionnaire (SRQ). The sampling technique not mentioned	Postpartum women (till 6 months of delivery) 16 or older Total participants = 175 Adolescent sample (16–19 years): n = 39	Adolescent (16–19):5 (14.7%) had clinical postpartum depression. Crude and adjusted odds of meeting clinical depression predicted by food insecurity among adolescents (16–19) vs. young (20–29): OR=0.898 (0.263-3.063) p = 0.25
Musyimi et al., 2020	Kenya Income level: Lower middle income Region: Sub-Saharan Africa	To investigate the factors associated with suicidal behavior among adolescent pregnant mothers in Kenya.	Socio-ecological model	Qualitative Interviews and focus group interviews. Purposive and snowball sampling	adolescent mothers, 15–19 years) n = 21 8 Key Informant Interviews were also interviewed	Poverty, intimate partner violence (IPV), family rejection, social isolation and stigma from the community, and chronic physical illnesses were associated with suicidal ideations.
Nasreen et al., 2011	Bangladesh Rural Income level: Lower middle income Region: South Asia	To estimate the prevalence of depressive and anxiety symptoms and explore the associated factors in a cross-section of rural Bangladeshi pregnant women.	None	Quantitative Cross-sectional data Edinburgh Postnatal Depression Scale (EPDS) Random Sampling	Women in their third trimester of pregnancy Total participants = 720 Adolescent sample (less than 19 years): n = 158	16.7% adolescent had antepartum depressive symptoms
Nicolet et al., 2021	Cameroon Urban Income level: Lower middle income Region: Sub-Saharan Africa	To identify and explore factors of perinatal depression among teenage mothers in Cameroon.	None	Quantitative Edinburgh Postnatal Depression Scale Sampling strategy not mentioned	Women in perinatal period, less than 20 years n = 1344	The prevalence of depressive disorder symptoms among teenage or young pregnant women is estimated to be 70.0%. This risk is significantly increased by different factors including unintended or unplanned pregnancy, experiencing depression and anxiety before childbirth, and domestic violence.
Nurbaeti et al., 2023	Indonesia Income level: Lower middle income Region: East Asia Pacific	To address the mental health and well-being of postpartum mothers in Indonesia, especially among adolescents	None	Quantitative Cross-sectional The Edinburgh Postnatal Depression Scale Cluster sampling	Muslim adolescent mothers in the postpartum period ranging, less than 20 years. n = 203	35.96% of teenage mothers experienced symptoms of postpartum depression. Marriage satisfaction, education level, family income, number of children, and baby weight at birth were significantly associated with postpartum depression. However, social support and religiosity showed no significant association with postpartum depression.
Okine et al., 2020	Ghana Mamprobi Polyclinic, Accra Income level: Lower middle income Region: Sub-Saharan Africa	To explore challenges faced by teenage mothers with repeat pregnancy and the support services available to them.	Bronfenbrenner’s (1979) ecological systems theory	Qualitative In-depth interviews &focus group discussions Convenience and Snowball sampling	Teenage mothers, less than 20 years Also included Health care workers n = 33 teenage and 8 HCW	Challenges include educational, financial, and health challenges. Adolescents experienced psychological issues, particularly stigma. They had feeling feelings of shame, anger and rejection, depression, and suicidal ideations. stigma was an issue they had to deal with daily.
Okunola et al., 2022	Nigeria semi-urban community Sampling technique not mentioned. Income level: Lower middle income Region: Sub-Saharan Africa	To evaluate the relationship between antenatal depression (APD) and postpartum depression (PPD) and predictors of PPD among an obstetric population in South-Western Nigeria.	None	Quantitative a prospective longitudinal cohort study Edinburgh Postnatal Depression Scale (EPDS) Sampling strategy not mentioned	Pregnant women between 34–36 weeks (follow-up up till 6 weeks of delivery) Total participants = 272 Adolescent sample (15–24 years): n = 29	15-24 years: 27.6% were depressed
Osok, Kigamwa, Huang, et al., 2018	Nairobi Kenya Health facility antenatal service Income level: Lower middle income Region: Sub-Saharan Africa	To elicit various practical, psychological, interpersonal, and cultural barriers to life adjustment, service access, obtaining resources, and psychosocial support related to pregnancy.	None	Qualitative Grounded theory In-depth semi-structured interviews (engagement interview approach) Purposive sampling	first-time Pregnant adolescents, 15-19 years n = 12	Challenges, including depression, anxiety and stress around the pregnancy, denial of the pregnancy, lack of basic needs provisions and care, and restricted educational or livelihood opportunities for personal development post pregnancy. These challenges led to negative mental health consequences in adolescent pregnant girls, including feeling insecure about the future, feeling very defeated and sad to be pregnant, and feeling unsupported and disempowered in providing care for the baby.
Osok, Kigamwa, Stoep, et al., 2018	Nairobi, Kenya urban resource-deprived areas Income level: Lower middle income Region: Sub-Saharan Africa	To determine the prevalence of depression and related psychosocial risks among pregnant adolescents reporting at a maternal and child health clinic in Nairobi, Kenya.	None	Quantitative Cross-sectional HQ-9 to assess depression. Convenient sampling	Pregnant adolescents, 15-18 years n = 176	2.9% (n = 58) had antenatal depression. Predictors include stressful life events, caregiver burden, absence of social support, being diagnosed with HIV/AIDS and being young.
Puey, 2022	Philippines colleges/University Income level: Lower middle income Region: East Asia & Pacific	To explore young mothers lived experiences and challenges at the University of Southern Mindanao.	None	Qualitative phenomenology Structured In-depth Interview Purposive sampling	Teenage mothers, 24 years or younger n = 25	Adolescent had psychological challenges including felt rejected, embarrassed and had broken relationship.
Putri., 2023	Indonesia Urban and rural Income level: Lower middle income Region: East Asia Pacific	To assess postpartum depression symptoms in young mother	None	Quantitative Cross-sectional survey Mini International Neuropsychiatric Interview (MINI) instrument Systematic linear sampling	Young mothers with babies less than 6 weeks, 15–24 years n = 1,285	The prevalence of postpartum depression in the 6 months postpartum was 4.0%, with a higher prevalence in urban areas (5.7%) than in rural areas (2.9%). Post partum depression was associated with living without a husband, experiencing preterm birth, having pregnancy complications, unwanted pregnancy, and having postpartum complications were associated with a higher risk of postpartum depression.
Taylor Salisbury et al., 2021	Mozambique, in the District of Manhiça Income level: Low income Region: Sub-Saharan Africa	To understand the experiences, causes, and priorities challenges that affect the mental health of young Mozambican mothers during the perinatal Period, and to co-design possible interventions	None	Qualitative Human centered design approach Focus-group discussions, individual interviews, and observations. Sampling technique not mentioned	Women who were pregnant or given birth within a year, 16-24 years n = 23 women Also included 12 family members, 19 service providers and 11 staff from the Ministry of Health.	Uncertainty focused on living situations, pregnancy outcomes, parenting, education, and financial stability, social support and limited knowledge. Several (39%) were happy about their pregnancy and felt emotionally and materially supported. Those with unplanned pregnancies, had mixed or negative feelings towards the pregnancy and the impact.
Tele et al., 2022	Nairobi Kenya Income level: Lower middle income Region: Sub-Saharan Africa	To find prevalence of depression and its associated risk factors among pregnant adolescents in Nairobi, Kenya	None	Quantitative cross-sectional Patient Health Questionnaire 9 Purposive sampling	pregnant adolescent, 14–18 years n = 153	43.1% of the respondents were depressed. Depressive symptoms in were independently associated with being in school, experience of intimate partner violence, substance use within the family and having experienced pressure to use substances by family or peers.
Tembo et al., 2023	Malawi Rural Income level: Low income Region: Sub-Saharan Africa	To identify the prevalence and social and cultural influences of depression among adolescents mothers attending community clinics at Mitundu in Lilongwe, Malawi.	None	Quantitative Cross-sectional Edinburgh Postnatal Depression Scale Convenience sampling	Adolescent postnatal mothers. Less than 19 years n = 395	43.6% (n = 172) presented with Postnatal depression (PND). Adolescents who had ever experienced intimate partner violence (IPV) were 13.6 times more likely to report PND, Participants whose families did not decide for them (regarding their care) were 2.3 times more likely to present with PND than those whose families, adolescent mothers who had interacted with their health worker were less likely to report PND than those who had no interaction with the health worker.
Tinago et al., 2023	Zimbabwe Income level: Lower middle income Region: Sub-Saharan Africa	To test the effectiveness of a community-based peer support intervention to mitigate social isolation and stigma of adolescent motherhood in Harare, Zimbabwe	None	Quasi experimental Patient Health Questionnaire (PHQ-9) Purposive and Snowball sampling	Adolescent mothers, 14–18 years n = 183 (intervention group = 104 and control group = 79)	The intervention arm reported lower depressive symptoms and common mental disorders and higher overall, family, friends, and significant-other support, compared to control. The intervention arm felt more engaged with peers, knew who and where to turn to for help, and had coping, parenting and communication strategies to manage life challenges.
Tirgari et al., 2020	Iran (Kerman province) Health centres Income level: Lower middle income Region: Middle East & North Africa	To explore experiences of teen mothers with stress and stressors of early motherhood	None	Qualitative In-depth semi-structured interviews Purposive sampling	Teen mothers less than 19 years n = 18	Five categories: 1) storm of anxiety (s fear and worry, regret and helplessness, guilty and ashamed, depression, loneliness, and isolation) 2) wander identity (Conflict between maternal and adolescence roles Role strain) 3) an unaccompanied way, (Insufficient familial and health support) 4) unarmed combat, (Knowledge and skill insufficiency) 5) and a tired body (Increasing responsibilities related to motherhood)
Undie & Birungi, 2022	Kenya Income level: Lower middle income Region: Sub-Saharan Africa	To examine the experience of teenage pregnancy and the resultant psychosocial support needs from the perspectives of both pregnant/parenting girls and their own parents, who are typically expected to provide various forms of support.	None	Qualitative Descriptive case study design Secondary data from counselling notes Sampling strategy not mentioned	Pregnant or parenting girls, teenage n = 20	Pregnant/parenting girls showed evidence of psychological trauma as a result of their pregnancies. The sexual debut of many girls occurred in the context of sexual violence wide range of psychological problems and mental health issues described by pregnant and parenting girls, including depression, fear, suicidal ideation, insomnia, reduced self-esteem, and hopelessness,
Uzobo, 2022	Nigeria Bayelsa State (in the health centre and clinic) Income level: Lower middle income Region: Sub-Saharan Africa	To examine the prevalence and coping strategies of Postnatal Depression (PND) among mothers in Bayelsa, Nigeria.	None	Quantitative Cross-sectional Self-developed questionnaire and Edinburgh’s scale Purposive sampling	Women with babies between 1–6 weeks, 15-44 years Total participants = 345 Adolescent sample (15–19 years): n = 21	15-19 years: Of all mothers, 21 (16.2%) had mild depression Findings of coping strategies were not reported separately.
Vahidi et al., 2023	Iran Urban and sub-urban Income level: Lower middle income Region: Middle East & North Africa	To investigate relationship between childbirth experience and PTSD with maternal functioning in Iranian adolescent mothers.	None	Quantitative Cross-sectional PTSD Symptom Scale, Childbirth Experience Questionnaire 2.0, and Barkin Index of Maternal Functioning Census Sampling	Adolescent women with one month and a maximum of 3 months have passed since giving birth, 10-19 years n = 202	There was a statistically significant relationship between PTSD, childbirth experience and unwanted baby sex with maternal functioning.
Wainaina et al., 2021	Nairobi, Kenya Income level: Lower middle income Region: Sub-Saharan Africa	To generate an inventory of mental stressors during pregnancy and early motherhood; understand how mental stress affects the ability to seek care for themselves and their child and understand individual coping strategies.	None	Qualitative Interviews and discussions from visual methodologies including Photovoice, digital storytelling, and public service announcements Sampling strategy not mentioned.	Pregnant and adolescent mothers, 14–19 years n = 30	The psychosocial challenges identified in order of importance included: chased from home by the parents; economic hardship; neglect and abandonment by the person responsible for the pregnancy; stigmatization by family, friends, and the community; feelings of shattered dreams; and daily stress related to living in poor and unhygienic conditions. During the pregnancy and early motherhood, the participants experienced feelings of embarrassment, shame, hopelessness, and to the extreme, suicidal thoughts clouded their minds. Main coping strategies included social isolation for some, socializing with other pregnant and adolescent mothers, and negative behaviors like the uptake of illicit drugs and alcohol and risky sexual relationships.
Webb et al., 2023	Uganda Income level: Low income Region: Sub-Saharan Africa	To explore the particular mental health impact of unmarried pregnancy among young rural Ugandan girls.	None	Qualitative ethnographic approach Fieldwork, group meetings, visual artifacts, and written records Sampling strategy not mentioned	Teenage mothers, 15-21 years n = 47	Mental health symptoms include isolation and social withdrawal, disruptive or disorganised behaviour, overthinking, substance use, sense of guilt or self-blame, anger and strong emotions, helplessness, grief. Poverty, gender inequality, patriarchy, poor family support, Community and family views (stigma and shame) were found to be contributors. Barriers to service utilization include attitudes, traditional/religious beliefs, and poor infrastructure.
Woollett et al., 2021	Zimbabwe Rural Income level: Lower middle income Region: Sub-Saharan Africa	To understand pregnant adolescent and young mothers in rural Zimbabwe by describing its risk profile and providing contextually meaningful suggestions for intervention design and implementation	None	Quantitative Self-administered tablet-administered questionnaires concerning maternal and child health, sexual and reproductive health, psychosocial well-being, and parenting Purposive sampling	Pregnant and young mothers 14-24 years n = 442	Psychological well-being: Twenty-four percent of adolescents reported that they often or always feel like they do not have much to be proud of. Sixty-nine percent of children reported that they never or only sometimes count on their friends when they need help. Forty percent reported never having lots of friends, while 16% reported that they are often or always very sad. It is positive to note that 64% of respondents felt that they always or often had at least one adult that loved them and that 63% always or often felt safe at home. Girls reported inadequate social support amidst high caretaking responsibilities and change in relocation for marriage, compromising mental health. Most of the pregnancies were unintended (approximately 60%), which had consequences on attachment and parenting, where roughly 40% of reported difficulties and lack of enjoyment in caring for their babies.
Yako, 2007	Lesotho hospital, health centres, and high school Income level: Lower middle income Region: Sub-Saharan Africa	To compare perceived stress in general, stress between a group of unmarried adolescent first-time mothers and a group of married adolescent first-time mothers.	None	Quantitative Cross-sectional comparative and descriptive Daily Hassles 53-Item Scale Developed by Kanner, Coyne & Lazarus (1981), Feeling of pregnancy Questionnaire (FOPQ) 78-item Scale Developed by Glazer (1979), and Postpartum Complication Checklist Convenience Sampling	Adolescent mothers 6 weeks post delivery 15-19 years n = 192 (dyads of 64 unmarried adolescent mothers and their infants, 64 married adolescents and their infants, and 64 high school students)	There were significant differences in perceived stress between both groups of adolescent mothers and the group of never-pregnant adolescents (p < .0001). Never-pregnant adolescents had the lowest levels of perceived stress (p < .0001). Both groups of adolescent mothers had high levels of stress due to pregnancy, and the difference between the two groups was non-significant.
Yoosefi Lebni et al., 2020	Iran, health centers of Kermanshah and Kurdistan Provinces Income level: Lower middle income Region: Middle East & North Africa	To explore the causes and grounds of childbirth fear and the strategies used by pregnant adolescent women in Iran to overcome such fears.	None	Qualitative semi-structured interviews Purposive sampling	Primiparous women, under 18 years n = 15	Participants reported fear of childbirth with subcategories of fear of child health, fear of childbirth process, fears about inappropriate medical staff performance, fears about hospital environment, and postpartum fears. strategies to reduce childbirth fear with subcategories of choosing appropriate medical centers, increasing information on childbirth, avoiding stressful sources, improving self-care, getting prepared for delivery day in advance and resorting to spirituality.

Abbreviations: AOR, adjusted odds ratio; CIDI, composite international diagnostic interview; COR, crude odds ratio; EPDS, edinburgh postnatal depression scale; FI, food insecurity; FOPQ, feeling of pregnancy questionnaire; GHQ, general health questionnaire; HCW, health care worker; HFIAS, household food insecurity access scale; HQ-9, health questionnaire-9; MINI, mini international neuropsychiatric interview; PHQ-9, patient health questionnaire-9; PND, postnatal depression; PPD, postpartum depression; PTSD, post-traumatic stress disorder; SRQ-20, self-reporting questionnaire-20; USAID, United States agency for international development; WHO, World Health Organization.

### Data analysis

Descriptive statistics, including frequencies and percentages of study characteristics and their findings, were summarised in a tabular manner. Narrative descriptions were employed to summarize features of studies that describe how the results connect to the review’s aim and questions. The presentation of these findings was further facilitated through the incorporation of tables.

## Results

**Fig 1:**
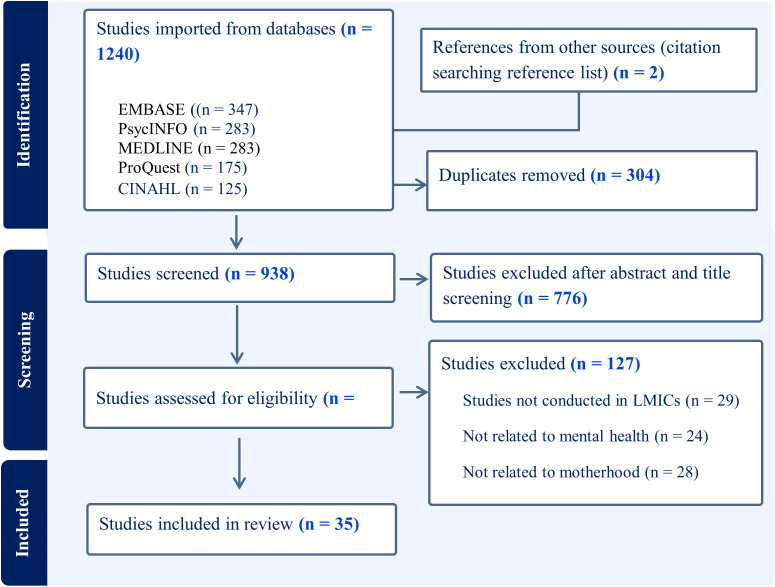
PRISMA-ScR flow diagram describes the screening process following the PRISMA-ScR guidelines. The flowchart illustrates the number of records identified through database and other sources, screened, assessed for eligibility, and finally included in the review, with reasons for exclusions.

1,240 articles were identified through database searches, supplemented by the identification of two additional articles through the process of citation searching reference lists. After removing 304 duplicates using Covidence, the remaining 938 records underwent screening based on titles and abstracts. Subsequently, 162 records were subjected to full-text screening. Applying the predetermined inclusion criteria, 35 records met the criteria and were included in the scoping review, as illustrated in the flowchart according to the Preferred Reporting Items for Systematic Reviews and Meta-Analyses for Scoping Reviews (PRISMA-ScR) (Fig 1).

### Study characteristics

More than half of the studies in this review (60%, n = 21) utilized quantitative designs [6,12,17,30–47], while 13 (37.1%) employed qualitative approaches [48–60], and one (2.9%) used an interventional design [61]. Among these studies, 13 (37.1%) had a sample size below 100, 14 (40%) had a sample size between 100 and 500, four studies (11.4%) had a sample size ranging from 500 to 1000, and four (11.4%) had a sample size exceeding 1000. Among 35 included articles, publication years spanned from 2007 to 2023, with 28 (80%) of the records being published within the last five years. All the studies covered the populations between the ages of 10 and 19 years.

### Countries of origin

The majority of these studies were from Sub-Saharan Africa (65.7%, n = 23) [17,31,32,34,37,39,41,42,44,45,47,48,50–53, 55,57–59,61–63]. Nairobi presented the highest evidence among other countries (20%, n = 7) [17,34,42,51,53,57,58], followed by Nigeria [41,45,50], Malawi [31,37,44], and Uganda [48,52,59] each constituting 8.6% (n = 3). Ethiopia [32,62] and Zimbabwe [47,61], each contributed two studies (5.7%). Studies from the Middle East and North Africa were limited to Iran (8.6%, n = 3) [46,56,60]. In South Asia, there was some evidence (11.4%, n = 4) [30,33,36,38], featuring varying counts across its listed countries, including studies from Bangladesh (5.7%, n = 2) [36,38], Sri Lanka (2.9%, n = 1) [30], and India (2.9%, n = 1) [33]. The East Asia and Pacific region contributed 14.3% (n = 5) of the evidence [35,40,43,49,54], with studies from the Philippines and Indonesia each with two studies (5.7%) [35,40,43,54] and one from Vietnam [49].

### Theoretical frameworks

Most studies (85.7%, n = 30) did not provide information on a theoretical framework. Among the studies that did provide a theoretical framework, three utilized Bronfenbrenner’s (1979) ecological systems theory (8.6%) [31,51,52], one employed the behavioral model [50], and one applied the stress and coping model [48].

The lack of theoretical framework was consistent across 20 quantitative studies, which primarily focused on measuring mental health outcomes or identifying correlations. In contrast, five studies informed by a theoretical framework included four studies that used qualitative designs [48,50–52] and one that used quantitative design [31]. Few studies acknowledged time constraints, limited training, or a focus on immediate public health priorities as challenges in incorporating theoretical frameworks. However, most studies did not discuss the reasons for the lack of a theoretical framework.

### Research focus on motherhood transition

Based on the findings of motherhood transitions across multiple phases (pregnancy, birth, and the postnatal period), the majority of studies (65.7%, n = 23) concentrated on the postnatal phase, while 19 studies (54.2%) focused on the antenatal period [17,30–32,34,36,38,39,42,47–49,52,53,55,57–59,63]. Only four articles (11.4%) delved into the labor and childbirth aspect [46,52,60,63]. Among these studies, few studies identified a combination of two phases [46–48,52,59,63] however, none of the studies covered all three phases: pregnancy, childbirth, and postnatal.

### Research focus on mental health

The majority of articles (n = 31, 88.6%) concentrated on mental health issues among adolescent mothers [17,30–48,52–60,62,63]. 20 studies (57.1%) investigated influences on mental health [17,31,33,34,36,37,39,40,42–44,46,51,53,55–60], three studies (8.6%) examined access and utilization of mental healthcare [35,50,59], while one article specifically targeted mental health intervention [61], and three studies (8.6%) explored coping strategies [48,58,60] (refer to Table 2). Out of the 35 articles, six (17.1%) [30,47,49,52,54,62], did not explicitly specify mental health as the primary focus of their study but included it as one of the sections discussing adolescents’ motherhood-related mental health challenges. While most studies examined individual foci separately within a specific study, a few studies explored the combined effects of multiple foci in a single study.

**Table 2:** Count of studies by mental health concept.

Outcomes of Studies related to Mental Health	Number of studies, n (%)
Mental health of adolescent mothers	31 (88.6)
Influences on mental health	20 (57.1)
Access and utilization of mental health care	3 (8.6)
Coping strategies	3 (8.6)
Interventions for improving mental health	1 (2.9)

*Note:* The sum does not add up to 100% as several studies have reported multiple outcomes.

### Impact of adolescent motherhood on mental health

The majority of articles (n = 31, 88.6%) focused on mental health issues that can be broadly grouped under mood disorders, anxiety disorders, and trauma responses [17,30–48,52–60,62,63]. Only one study identified a positive influence, specifically feelings of happiness and pride [49]. Among the 20 studies (57%) that explored factors influencing mental health, stressors included food insecurity, violence, lack of support, HIV/AIDS diagnosis, insufficient provisioning and care, social stigma, poverty, patriarchy, husband’s migration for employment, delivery by caesarean section, unplanned pregnancy, and challenges related to education, finances, and employment [17,31,33,34,36,37,39,40,42–44,46,51,53,55–60].

Additionally, three studies (8.6%) addressed mental healthcare access and utilization. Barriers identified included negative attitudes, traditional/religious beliefs, inadequate infrastructure, and distant health facility access [35,50,59]. These barriers hindered access to services and negatively influenced perceptions of care.

Coping mechanisms were found in three studies (8.6%), encompassing personal motivation, spirituality, seeking support, utilizing opportunities, choosing suitable medical centers, and enhancing childbirth knowledge [48,58,60]. Only one study was an interventional study incorporating Community-Based Peer Support that was found to be significant in decreasing depressive symptoms and common mental disorders [61].

### Discussion and future implications

In this scoping review, we identified 35 primary studies addressing adolescent mothers’ mental health across LMICs. These studies offer valuable insights into how motherhood affects adolescent well-being.

### Type and extent of research on mental health of adolescent mothers

#### Mental health of adolescent mothers.

Our review findings reveal that adolescents face an elevated risk of experiencing poor mental health [64,65]. There was substantial variability in the prevalence of antenatal depression across LMICs ranging from 16.7% in Bangladesh [38] to 70% in Cameroon [39] compared to an average of 12% in high-income countries [66,67]. Similarly, postpartum depression ranged from 18.8% in Burkina Faso [31] to 43.6% in Malawi [44], surpassing the global estimate of 17.2% [68].

Mental health issues in this population extend beyond depression. Substance use frequently co-occurs with depressive symptoms, as shown in a Nairobi study where 24.5% of depressed pregnant adolescents engaged in substance use [34]. Suicidal ideation affected 14.3% of pregnant adolescents in Ethiopia [32], with suicide attempts reported by 6.5% in Bangladesh [36]. These outcomes are linked to factors such as poverty, intimate partner violence, family rejection, stigma, and chronic illness [17,36,39,44,69].

Posttraumatic stress symptoms were reported in 18.3% of adolescent mothers in Iran [46], consistent with the literature that suggests higher levels of perceived stress among pregnant adolescents compared to their never-pregnant peers [63]. Studies found that adolescent mothers view childbirth as traumatic, shaped by experiences such as fear of death, loss of control, marital status, and sexual partner violence [17,44,70]. Fear of childbirth was also documented, involving concerns about the labor process, the hospital environment, the child’s health, and postpartum recovery [60]. These concerns reflect broader patterns observed in prior research, where adolescent mothers often felt unprepared for childbirth due to lack of experience and psychological readiness [71,72].

In addition, adolescent mothers frequently reported emotional distress, including worry, isolation, helplessness, guilt, shame, and regret [49,53,56,58,59]. These symptoms often reflect underlying anxiety, depression, and possible post-traumatic stress disorder [48,73].

Although most studies emphasize negative mental health outcomes, one study reported a positive experience, noting that some adolescent mothers felt happiness in fulfilling family roles and pleasing their husbands and extended families [49]. These positive emotions were attributed to strong social support, cultural validation of motherhood, and the perceived achievement of adult status. Such findings indicate that when adolescents receive affirmation and support from their immediate social environment, emotional resilience may be enhanced. These insights point to the value of strength-based approaches that not only mitigate risk but also foster protective factors rooted in local cultural contexts [18].

The disparities in mental health outcomes among LMICs reflect broader structural issues, including limited mental health infrastructure, shortages of trained professionals, and restricted access to services in rural areas [50]. Cultural stigma around adolescent pregnancy and mental illness further restricts help-seeking and recognition of psychological distress [59,74]. Additionally, the heterogeneity in prevalence rates may be attributed to methodological differences across studies, such as variations in sampling methods, study design, assessment tools, and screening tool cut-off points [66,75,76]. These inconsistencies highlight the need for more rigorous, harmonized approaches in future research to enhance comparability and inform policy development.

#### Influences on mental health.

Our review highlighted a broad range of factors influencing the mental health of adolescent mothers. Key determinants identified across the included studies include limited education [31,40,53,55], poverty [40,51,55,58,59], marital status [59], food insecurity [37], number of children [40], child weight [40], rigid social norms [59], and absence of social support [31,33,51,53,55,58,59]. These findings align with existing literature, suggesting that adolescent mothers are more likely to reside in low-income communities, be born to parents with limited educational and employment achievements, have a history of child abuse, live in turbulent home environments marked by strained interpersonal relationships, and have restricted social support networks [18,77]. Collectively, these conditions are associated with increased vulnerability to adverse mental health outcomes.

Additional stressors included unintended pregnancy, pregnancy complications, and the gender of the child [33,39,43,46,51]. Unplanned pregnancies were commonly linked to increased depression, anxiety, and emotional distress among adolescent mothers [39,47]. These challenges were worsened by limited readiness for parenthood, strained family ties, and fears of disrupted life plans [39]. Some also struggled to bond with their infants, as adolescent mothers faced difficulty forming emotional attachments and lacked fulfillment in the caregiving role [47]. This burden intensified in gender-biased contexts. A study from India reported that young women who delivered female infants faced 2.43 times higher odds of distress compared to those with male infants [33]. This finding aligns with earlier research conducted in South Asia, indicating that women undergo psychological distress following the birth of a female child due to entrenched gender norms that favor sons [78–82]. Such norms and discriminatory practices represent violations of adolescent mothers’ rights to health, dignity, and protection, as outlined in international human rights frameworks [16,79].

Inadequate support also emerged as significant stressor for adolescent mother’s mental health [31,51,52,59]. The lack of support from spouse, family, and healthcare providers contributes to elevated stress levels. Support plays a crucial role in mitigating depression and mental health problems. Struggle to balance maternal responsibilities and the pursuit of competencies essential for effective motherhood further compounds their stress and contribute to identity conflict [63].

Insufficient knowledge and skills, particularly in areas such as pregnancy and delivery care, breastfeeding, childcare, health behaviors, and accessing supportive resources, contribute to their stress [55,56]. These stressors are further intensified by stigma originating from immediate and extended family members, particularly in cases of unmarried or repeatedly pregnant adolescents, who are often perceived as having brought dishonor to their families [58].

HIV-positive status emerged as an additional risk factor [53]. Depression and stigma associated with HIV during pregnancy can hinder the HIV care and adherence to antiretroviral therapy (ART). This is consistent with broader literature suggesting that internalized HIV-related stigma can compound depression during pregnancy [83]. Recognizing these multifaceted stressors is crucial for developing comprehensive interventions and support systems for adolescent mothers facing mental health challenges. However, while these associations were consistently reported, the depth of analysis varied across studies. Few studies identified correlations without exploring causal mechanisms or adequately controlling for confounding variables, which limits the generalizability and robustness of the findings.

#### Coping mechanism used by adolescent mothers.

The review highlighted coping strategies employed by adolescent mothers in LMICs. Some of the adolescents demonstrated resilience, fueled by a desire to succeed and a positive outlook on the future [48]. Such coping strategies included pursuing education and vocational skills. Faith and religion also emerged as significant coping mechanism, helping them avoid overthinking about their situations [60]. Notably, comparable coping strategies have been identified in previous studies, suggesting commonalities in resilience strategies across different contexts [74,84]. However, in some contexts, religious framing of suffering as a test of faith or morality may suppress open discussion of abuse or deter help-seeking, particularly in cases of sexual violation [52,59]. Some mothers relied on the support of family, friends, and spouse, while others exhibited adaptability after childbirth, attributing their focus to lessons learned and an enhanced understanding of the world [60]. Comparable findings highlighted the complexity of managing stress during this pivotal life transition [85]. However, it is important to consider that not all adolescents cope equally; some face stigma, express regret, and engage in negative behaviors like isolation, risky relationships, and drug use [58].

#### Access and utilization of mental health service.

The scoping review revealed obstacles and support for adolescent mothers accessing mental health services. Perceived benefits of seeking care, such as motivation to address depressive symptoms, positive interactions with providers, and access to informational support were cited as facilitators [50]. However, social and cultural norms, as discussed in previous studies, create hesitancy and fear of judgment, hindering service utilization [50,52,58,59]. Stigmatization, particularly by health providers surrounding both mental health issues, early pregnancy, single motherhood and unwed pregnancies often discourages individuals from seeking help, especially in communities where discussions about mental health are restricted. The social and cultural norms may also lead to blaming young mothers for their situation, which makes them less likely to use mental health services. Additionally, traditional and religious beliefs add to this stigma, with some cultural contexts viewing mental health struggles as a sign of personal weakness or divine punishment [52,59]. These deeply rooted attitudes cause adolescent mothers to be hesitant about seeking help, fearing judgment or isolation [74]. These barriers are not just cultural, they represent systemic violations of adolescent girls’ right to access timely, appropriate, and non-discriminatory mental health care.

Another significant barrier was the limited access to health facilities for adolescent mothers in remote areas [59]. Geographic constraints and transportation difficulties often prevent individuals from reaching mental health facilities, especially when these facilities are concentrated in urban areas. A shortage of mental health care workers and facilities primarily located in urban areas also exacerbates the challenges of accessing services for adolescent mothers [59]. These access issues are particularly acute in LMICs, where mental health funding is low and resources are disproportionately distributed [10]. Adolescent mothers are particularly burdened, as they often face encounter additional barriers, including limited mobility due to economic or familial dependency, social stigma surrounding early motherhood, and reduced autonomy in decision-making [1,2]. These disparities further restrict access to mental health services, making it challenging for adolescent mothers in underserved areas to receive timely and appropriate mental health support.

#### Mental health interventions for adolescent mothers.

Our review identified only one interventional study incorporating Community-Based Peer Support. In the identified intervention, participants attended bi-monthly face-to-face sessions (12 total, 75 minutes each), with WhatsApp used for coordination, Q&A, and continued engagement. This approach led to a reduction in depressive symptoms and common mental disorders [61]. A comparable study conducted in South Africa echoed these findings, highlighting the benefits of peer group support in fostering emotional connection and meaningful social networks among adolescent mothers [86]. Sanders et al. (2022) further advocate for the integration of school- and community-based parenting programs to improve outcomes for young parents [87].

In light of findings from the review, we propose strategies for improving the mental health of adolescent mothers in LMICs.

- Establish structured community-based peer support groups where adolescent mothers meet regularly for guided discussions led by trained peers [61].
- Design mobile-based mental health tools (e.g., SMS or app-based counseling) that are low-cost and accessible, allowing adolescent mothers to receive emotional support and reduce stigma-related isolation, particularly in low-resource settings where mental health services are limited [61].
- Educate frontline healthcare workers to identify signs of emotional distress, suicidal ideation, and trauma among adolescent mothers using trauma-informed care principles, while fostering nonjudgmental attitudes [18,50].
- Implement targeted family and partner involvement programs that aim to reduce interpersonal violence, increase male engagement in postnatal care, and address underlying gender norms affecting adolescent motherhood and mental health [14].
- Integrate mandatory mental health screening and referral services into routine antenatal and postnatal care, using simple, validated screening tools adapted for adolescents [32].
- Design educational and vocational programs targeting adolescent mothers that not only improve skills and employability but also build self-esteem and resilience [8].
- Secure policy-level support and cross-sector collaboration, ensuring that ministries of health, education, and social welfare co-design and implement adolescent-responsive mental health programming [14].

### Gaps in literature

The following gaps were identified:

#### Methodological.

The predominant emphasis on quantitative studies in this review highlights gaps in existing literature. Motherhood’s impact is highly individualized and challenging to quantify [88]. While quantitative methods are valuable for measuring mental health outcomes, their limitations in providing contextual understanding emphasize the importance of qualitative studies. The review findings highlight the need for context-specific qualitative studies to capture the diverse, culturally grounded experiences of adolescent motherhood.

#### Geographical.

The review analyzed 35 studies from 17 countries, which accounted for 13.1% of the total 137 LMICs. Specifically, only four studies were from South Asia, constituting 10% of the total 30 countries in that region, and no studies were identified from Latin America and the Caribbean, East Asia and Pacific, or Europe and Central Asia. These results indicate a constrained research landscape regarding this topic in LMICs. Possible contributing factors include structural inequities in global research funding, language barriers in publication, and institutional research capacities [89,90]. For instance, mental health research in Latin America and the Middle East is often marginalized due to linguistic exclusion and limited integration into international research agendas [6,72]. Additionally, contextual factors may influence the shaping of research priorities in these regions. For example, within the South Asian context, adolescent motherhood is often disguised by prevalent societal norms and cultural expectations, making it a silent struggle for young mothers who face limited access to healthcare and support systems [89]. The deeply rooted cultural taboos surrounding premarital pregnancies and early marriages [91] further exacerbate the challenges faced by adolescent mothers, hindering open discussions and comprehensive research on their mental health needs. Future funding should support research, potentially in all regions and countries, to understand the impact of adolescent motherhood on mental health.

#### Theoretical framework.

A lack of theoretical frameworks in the included studies emerged as a gap, which limits the ability to contextualize results, compare outcomes across contexts, and design culturally grounded mental health interventions for adolescent mothers in LMICs. Given the intricate nature of adolescent motherhood and its impact on mental health, incorporating theoretical foundations becomes essential to develop comprehensive and responsive strategies [92]. While the challenges of motherhood in isolation may contribute to negative mental health conditions, the intersection of this role with other socio-cultural identities (such as race, ethnicity, gender identity, sexual orientation, socioeconomic status) elicits distinctive circumstances that need to be considered. One promising theoretical framework for delving into the mental health experiences of adolescent mothers is the intersectionality perspective, which can be applied in future research to examine how overlapping identities such as age, gender, socioeconomic status, and cultural background collectively influence mental health outcomes. Researchers could use intersectional analysis to disaggregate data by multiple identity markers and explore how structural inequalities affect access to mental health services across different subgroups [93]. This lens can help uncover unique psychosocial stressors or barriers to care that arise from compounded forms of marginalization, thereby informing targeted interventions and culturally responsive policies [92]. Longitudinal or comparative studies using intersectional frameworks across varied LMIC settings could further illuminate context-specific disparities and resilience factors.

Another theoretical framework to consider is transition theory, which elucidates transition as periods of instability and vulnerability. Applying this framework in future studies could help researchers systematically examine the psychological and social shifts that adolescent mothers experience, from pregnancy through postpartum [94]. Future research might use this theoretical framework to map out stages of transition and identify both risk and protective factors influencing mental health at each phase. In LMIC contexts especially, the theoretical framework can guide culturally sensitive, stage-specific mental health strategies that address transitional stressors unique to adolescent populations [94].

In addition, the socioecological framework (which emphasizes multi-level influences from individual to policy level) and life course theory (which considers long-term effects of early exposures) can enable more holistic and equity-oriented approaches [23,78,79]. These theoretical frameworks can structure future studies to explore how community, familial, and institutional factors shape adolescent mothers’ mental health over time and help align research with appropriate mental health programs in LMIC settings.

#### Motherhood and mental health.

The review found that studies predominantly addressed individual transitional phases—antenatal, birth, and postnatal without extensively exploring the interactions and interdependencies between these phases. Only a limited number of studies (20%) consider both antenatal and postnatal periods, while just one study incorporates all three phases. This lack of integration between phases overlooks the continuum of motherhood experiences and the potential cumulative impact of multiple transitions on a woman’s journey into motherhood. Previous research suggests that an integrated approach to studying motherhood transitions, encompassing antenatal, birth, and postnatal phases, can provide a more nuanced understanding of the dynamic processes involved [4]. The interconnectedness of these phases highlights the need for a comprehensive perspective to capture the evolving nature of motherhood experiences. Addressing this gap in literature would contribute significantly to the existing knowledge base and facilitate a more holistic understanding of the complexities inherent in the transition to motherhood.

Further, the predominant focus of the studies was on mental health issues, particularly emphasizing mental health disorders and their symptoms among adolescents. Nevertheless, a notable gap exists in the research landscape, as there is a scarcity of studies exploring the predictors of mental health issues, barriers to the utilization of mental healthcare, mental health interventions, and coping strategies in this population. This gap suggests a need for a more comprehensive understanding of the factors influencing mental health and the resources available to address it among adolescent mothers. Furthermore, it was observed that certain studies did not explicitly designate mental health as a primary focus but rather included it as a subsection within the broader context of adolescents’ experiences related to motherhood. This observation highlights the importance of integrating mental health considerations more explicitly into research on adolescent motherhood, as mental well-being plays a critical role in their overall health and resilience. Recommendations include fostering more research efforts to investigate stressors and coping strategies for mental health outcomes, and urging a more integrated approach to studying mental health within the specific context of adolescents’ motherhood-related experiences [95].

### Strengths and limitations

The review’s strength lies in its rigorous methodology, which included a clearly defined eligibility criteria, a detailed results summary, and independent screening at all stages of the study selection process. While the current scoping review provides valuable insights, it has certain limitations that could be addressed in future research. First, the methodological quality of the included studies was not appraised, consistent with the optional nature of quality appraisal in scoping reviews. Nevertheless, this limits our ability to evaluate the strength or credibility of the evidence base. Future reviews may consider incorporating quality appraisal to enhance analytical rigor. Second, the restriction to English-language studies, may have introduced selection bias, potentially omitting culturally relevant evidence from non-English speaking LMICs. While this could have affected the geographic diversity of included studies, the overall findings remained consistent across diverse LMIC settings. Third, the optional sixth stage of Arksey and O’Malley’s (2005) scoping review framework stakeholder consultation was not conducted due to time and resource constraints [24]. While, this may have reduced opportunities to validate or contextualize the findings with practitioner and community insights, the study still provides a valuable synthesis grounded in empirical literature. Incorporating stakeholder input in future reviews could help contextualize findings and enrich interpretation. Lastly, although some studies included participants up to age 24, data specific to adolescents (10–19) were extracted where possible. While some developmental overlaps exist, their impact on the synthesis is likely minimal.

## Conclusion

This scoping review synthesized evidence from 35 studies across LMICs, revealing a substantial burden of mental health challenges among adolescent mothers. Key mental health concerns included depression, anxiety, suicidality, PTSD, and emotional distress. These challenges are driven by structural and interpersonal factors such as poverty, stigma, gender norms, lack of support, and inadequate access to mental health services.

The evidence base is dominated by quantitative studies with minimal theoretical guidance, limited exploration of coping strategies, and only one interventional study. Most research focused on postnatal periods, neglecting the antenatal and childbirth phases, and failed to consider the full motherhood continuum.

The findings highlight policy and practice needs: integrating adolescent-specific mental health screening and counseling into routine maternal care; scaling up community-based peer support and mobile health interventions; and training healthcare providers in adolescent-responsive, trauma-informed care. Future research should adopt approaches informed by theoretical frameworks, along with longitudinal and qualitative methods, particularly in underrepresented regions, to inform context-specific, effective interventions.

## Supporting information

S1 Checklist PRISMA-ScR Checklist. (DOCX)

S1 File Search strategy. (DOCX)

S2 File Data extraction table. (DOCX)

## References

[1] Mangeli M, Rayyani M, Cheraghi MA, Tirgari B. Exploring the Challenges of Adolescent Mothers From Their Life Experiences in the Transition to Motherhood: A Qualitative Study. J Family Reprod Health. 2017;11(3):165–73. 30018654 PMC6045691

[2] Crooks R, Bedwell C, Lavender T. Adolescent experiences of pregnancy in low-and middle-income countries: a meta-synthesis of qualitative studies. BMC Pregnancy Childbirth. 2022;22(1):702. doi: 10.1186/s12884-022-05022-1 36096763 PMC9469636

[3] Fouquier KF. The concept of motherhood among three generations of African American women. J Nurs Scholarsh. 2011;43(2):145–53. doi: 10.1111/j.1547-5069.2011.01394.x 21605318

[4] Mercer RT. The Process of Maternal Role Attainment over the First Year. Nurs Res. 1985;34(4):198???203. doi: 10.1097/00006199-198507000-00002 3847870

[5] Uriko K. Dialogical Self and the Changing Body During the Transition to Motherhood. J Const Psychol. 2018;32(3):221–35. doi: 10.1080/10720537.2018.1472048

[6] Yopo Díaz M. Enacting motherhood: time and social change in Chile. J Gend Stud. 2016;27(4):411–27. doi: 10.1080/09589236.2016.1223619

[7] Razurel C, Bruchon-Schweitzer M, Dupanloup A, Irion O, Epiney M. Stressful events, social support and coping strategies of primiparous women during the postpartum period: a qualitative study. Midwifery. 2011;27(2):237–42. doi: 10.1016/j.midw.2009.06.005 19783333

[8] van Vugt E, Versteegh P. “She gave me hope and lightened my heart”: The transition to motherhood among vulnerable (young) mothers. Child Youth Serv Rev. 2020;118:105318. doi: 10.1016/j.childyouth.2020.105318

[9] World Health Organization. Safe motherhood day 2020. 2020. Available from: https://www.who.int/bangladesh/news/detail/27-05-2020-safe-motherhood-day-2020

[10] World Health Organization. Adolescent pregnancy. 2022. Available from: https://apps.who.int/iris/bitstream/handle/10665/112320/WHO_RHR_14.08_eng.pdf

[11] Chung HW, Kim EM, Lee J-E. Comprehensive understanding of risk and protective factors related to adolescent pregnancy in low- and middle-income countries: A systematic review. J Adolesc. 2018;69:180–8. doi: 10.1016/j.adolescence.2018.10.007 30390598 PMC6284104

[12] Kassa GM, Arowojolu AO, Odukogbe AA, Yalew AW. Prevalence and determinants of adolescent pregnancy in Africa: a systematic review and Meta-analysis. Reprod Health. 2018;15(1):195. doi: 10.1186/s12978-018-0640-2 30497509 PMC6267053

[13] Cahyaningtyas DK, Astuti AW, Hani U. Parents involvement and barriers of programme interventions to reduce adolescent pregnancy. J Heal Technol Assess Midwifery. 2020;3(2):73–86. doi: 10.31101/jhtam.1312

[14] Kumar M, Huang K-Y, Othieno C, Wamalwa D, Madeghe B, Osok J, et al. Adolescent Pregnancy and Challenges in Kenyan Context: Perspectives from Multiple Community Stakeholders. Glob Soc Welf. 2018;5(1):11–27. doi: 10.1007/s40609-017-0102-8 29744286 PMC5937539

[15] Safdari-Dehcheshmeh F, Noroozi M, Taleghani F, Memar S. Factors Influencing the Delay in Childbearing: A Narrative Review. Iran J Nurs Midwifery Res. 2023;28(1):10–9. doi: 10.4103/ijnmr.ijnmr_65_22 37250942 PMC10215553

[16] UNICEF. Adolescent Health The Missing Population in Universal Health Coverage. 2020. Available from: https://www.unicef.org/media/58171/file

[17] Tele A, Kathono J, Mwaniga S, Nyongesa V, Yator O, Gachuno O, et al. Prevalence and risk factors associated with depression in pregnant adolescents in Nairobi, Kenya. J Affect Disord Rep. 2022;10:100424. doi: 10.1016/j.jadr.2022.100424 36970124 PMC10038142

[18] Hodgkinson S, Beers L, Southammakosane C, Lewin A. Addressing the mental health needs of pregnant and parenting adolescents. Pediatrics. 2014;133(1):114–22. doi: 10.1542/peds.2013-0927 24298010 PMC3876179

[19] Chauhan A, Potdar J. Maternal Mental Health During Pregnancy: A Critical Review. Cureus. 2022;14(10):e30656. doi: 10.7759/cureus.30656 36426343 PMC9681705

[20] Ganchimeg T, Ota E, Morisaki N, Laopaiboon M, Lumbiganon P, Zhang J, et al. Pregnancy and childbirth outcomes among adolescent mothers: a World Health Organization multicountry study. BJOG Int J Obstet Gynaecol. 2014;121:40–8. doi: 10.1111/1471-0528.12630 24641534

[21] Grønvik T, Fossgard Sandøy I. Complications associated with adolescent childbearing in Sub-Saharan Africa: A systematic literature review and meta-analysis. PLoS One. 2018;13(9):e0204327. doi: 10.1371/journal.pone.0204327 30256821 PMC6157872

[22] Duby Z, McClinton Appollis T, Jonas K, Maruping K, Dietrich J, LoVette A, et al. “As a Young Pregnant Girl… The Challenges You Face”: Exploring the Intersection Between Mental Health and Sexual and Reproductive Health Amongst Adolescent Girls and Young Women in South Africa. AIDS Behav. 2021;25(2):344–53. doi: 10.1007/s10461-020-02974-3 32683636 PMC7368608

[23] Mutumba M, Harper GW. Mental health and support among young key populations: an ecological approach to understanding and intervention. J Int AIDS Soc. 2015;18(2 Suppl 1):19429. doi: 10.7448/IAS.18.2.19429 25724505 PMC4344542

[24] Arksey H, O’Malley L. Scoping studies: towards a methodological framework. Int J Soc Res Methodol. 2005;8(1):19–32. doi: 10.1080/1364557032000119616

[25] Levac D, Colquhoun H, O’Brien KK. Scoping studies: advancing the methodology. Implement Sci. 2010;5:69. doi: 10.1186/1748-5908-5-69 20854677 PMC2954944

[26] World Bank. Lower middle income. 2020. Available from: https://data.worldbank.org/country/XN

[27] Better systematic review management. Covidence. 2025. Available from: https://www.covidence.org/

[28] Peters MDJ, Marnie C, Tricco AC, Pollock D, Munn Z, Alexander L, et al. Updated methodological guidance for the conduct of scoping reviews. JBI Evid Synth. 2020;18(10):2119–26. doi: 10.11124/JBIES-20-00167 33038124

[29] Tricco AC, Lillie E, Zarin W, O’Brien KK, Colquhoun H, Levac D, et al. PRISMA Extension for Scoping Reviews (PRISMA-ScR): Checklist and Explanation. Ann Intern Med. 2018;169(7):467–73. doi: 10.7326/M18-0850 30178033

[30] Agampodi TC, Wickramasinghe ND, Jayakodi HG, Amarasinghe GS, Warnasekara JN, Hettiarachchi AU, et al. The hidden burden of adolescent pregnancies in rural Sri Lanka; findings of the Rajarata Pregnancy Cohort. BMC Pregnancy Childbirth. 2021;21(1):494. doi: 10.1186/s12884-021-03977-1 34233652 PMC8265066

[31] Ajayi AI, Chamdimba E, Sawadogo N, Gitahi N, Tarnagda AM, Ilboudo AK, et al. Socio-ecological factors associated with probable depression among pregnant and parenting adolescent girls: findings from a cross-sectional study in Burkina Faso and Malawi. Reprod Health. 2023;20(1):38. doi: 10.1186/s12978-023-01588-x 36882850 PMC9990966

[32] Belete K, Kassew T, Demilew D, Amare Zeleke T. Prevalence and Correlates of Suicide Ideation and Attempt among Pregnant Women Attending Antenatal Care Services at Public Hospitals in Southern Ethiopia. Neuropsychiatr Dis Treat. 2021;17:1517–29. doi: 10.2147/NDT.S309702 34040377 PMC8140917

[33] Khanna T, Garg P, Akhtar F, Mehra S. Association between gender disadvantage factors and postnatal psychological distress among young women: A community-based study in rural India. Glob Public Health. 2021;16(7):1068–78. doi: 10.1080/17441692.2020.1820066 32928069

[34] Kimbui E, Kuria M, Yator O, Kumar M. A cross-sectional study of depression with comorbid substance use dependency in pregnant adolescents from an informal settlement of Nairobi: drawing implications for treatment and prevention work. Ann Gen Psychiatry. 2018;17:53. doi: 10.1186/s12991-018-0222-2 30598688 PMC6300883

[35] Labrague LJ, McEnroe-Petitte D, Tsaras K, Yboa BC, Rosales RA, Tizon MM, et al. Predictors of postpartum depression and the utilization of postpartum depression services in rural areas in the Philippines. Perspect Psychiatr Care. 2020;56(2):308–15. doi: 10.1111/ppc.12428 31355473

[36] Li J, Imam SZ, Jing Z, Wang Y, Zhou C. Suicide attempt and its associated factors amongst women who were pregnant as adolescents in Bangladesh: a cross-sectional study. Reprod Health. 2021;18(1):71. doi: 10.1186/s12978-021-01127-6 33789699 PMC8011090

[37] Mark TE, Latulipe RJ, Anto-Ocrah M, Mlongoti G, Adler D, Lanning JW. Seasonality, Food Insecurity, and Clinical Depression in Post-Partum Women in a Rural Malawi Setting. Matern Child Health J. 2021;25(5):751–8. doi: 10.1007/s10995-020-03045-8 33231821

[38] Nasreen HE, Kabir ZN, Forsell Y, Edhborg M. Prevalence and associated factors of depressive and anxiety symptoms during pregnancy: a population based study in rural Bangladesh. BMC Womens Health. 2011;11:22. doi: 10.1186/1472-6874-11-22 21635722 PMC3117808

[39] Nicolet L, Moayedoddin A, Miafo JD, Nzebou D, Stoll B, Jeannot E. Teenage Mothers in Yaoundé, Cameroon-Risk Factors and Prevalence of Perinatal Depression Symptoms. J Clin Med. 2021;10(18):4164. doi: 10.3390/jcm10184164 34575274 PMC8470336

[40] Nurbaeti I, Lestari KB, Syafii M. Association between Islamic religiosity, social support, marriage satisfaction, and postpartum depression in teenage mothers in West Java, Indonesia: A cross-sectional study. Belitung Nurs J. 2023;9(4):313–21. doi: 10.33546/bnj.2661 37645571 PMC10461155

[41] Okunola TO, Awoleke JO, Olofinbiyi B, Rosiji B, Olubiyi AO, Omoya S. Predictors of postpartum depression among an obstetric population in South-Western Nigeria. J Reprod Infant Psychol. 2022;40(4):420–32. doi: 10.1080/02646838.2021.1886259 33641549

[42] Osok J, Kigamwa P, Stoep AV, Huang K-Y, Kumar M. Depression and its psychosocial risk factors in pregnant Kenyan adolescents: a cross-sectional study in a community health Centre of Nairobi. BMC Psychiatry. 2018;18(1):136. doi: 10.1186/s12888-018-1706-y 29776353 PMC5960084

[43] Putri AS, Wurisastuti T, Suryaputri IY, Mubasyiroh R. Postpartum Depression in Young Mothers in Urban and Rural Indonesia. J Prev Med Public Health. 2023;56(3):272–81. doi: 10.3961/jpmph.22.534 37287205 PMC10248106

[44] Tembo C, Portsmouth L, Burns S. Postnatal depression and its social-cultural influences among adolescent mothers: A cross sectional study. PLOS Glob Public Health. 2023;3(6):e0002025. doi: 10.1371/journal.pgph.0002025 37352145 PMC10289363

[45] Uzobo E, Teibowei BJ, Ogeh VI. Prevalence and Coping Strategies of Postnatal Depression among Women in Bayelsa State, Nigeria. Afr J Nurs Midwifery. 2022;24(1). doi: 10.25159/2520-5293/9457

[46] Vahidi F, Mirghafourvand M, Naseri E, Ghanbari-Homaie S. Birth-related posttraumatic stress disorder and negative childbirth experience related to maternal functioning among adolescent mothers: a cross-sectional study. BMC Pregnancy Childbirth. 2023;23(1):371. doi: 10.1186/s12884-023-05717-z 37217921 PMC10201514

[47] Woollett N, Bandeira M, Marunda S, Mudekunye L, Ebersohn L. Adolescent pregnancy and young motherhood in rural Zimbabwe: Findings from a baseline study. Health Soc Care Community. 2021;29(6):e377–86. doi: 10.1111/hsc.13362 33825254

[48] Kaye DK. Negotiating the transition from adolescence to motherhood: coping with prenatal and parenting stress in teenage mothers in Mulago hospital, Uganda. BMC Public Health. 2008;8:83. doi: 10.1186/1471-2458-8-83 18318894 PMC2297507

[49] Klingberg-Allvin M, Binh N, Johansson A, Berggren V. One foot wet and one foot dry: transition into motherhood among married adolescent women in rural Vietnam. J Transcult Nurs. 2008;19(4):338–46. doi: 10.1177/1043659608322419 18669900

[50] Kola L, Bennett IM, Bhat A, Ayinde OO, Oladeji BD, Abiona D, et al. Stigma and utilization of treatment for adolescent perinatal depression in Ibadan Nigeria. BMC Pregnancy Childbirth. 2020;20(1):294. doi: 10.1186/s12884-020-02970-4 32410586 PMC7226964

[51] Musyimi CW, Mutiso VN, Nyamai DN, Ebuenyi I, Ndetei DM. Suicidal behavior risks during adolescent pregnancy in a low-resource setting: A qualitative study. PLoS One. 2020;15(7):e0236269. doi: 10.1371/journal.pone.0236269 32697791 PMC7375578

[52] Okine L, Dako-Gyeke M, Baiden P, Saa-Touh Mort K. Exploring the influence of repeat pregnancy on the lives of teenage mothers. J Hum Behav Soc Environ. 2020;30(7):863–80. doi: 10.1080/10911359.2020.1763226

[53] Osok J, Kigamwa P, Huang K-Y, Grote N, Kumar M. Adversities and mental health needs of pregnant adolescents in Kenya: identifying interpersonal, practical, and cultural barriers to care. BMC Womens Health. 2018;18(1):96. doi: 10.1186/s12905-018-0581-5 29902989 PMC6003032

[54] Pueyo J. Moms too soon: Status and challenges of teenage mothers. J Int Womens Stud. 2022;23(6).

[55] Taylor Salisbury T, Atmore KH, Nhambongo I, Mintade M, Massinga L, Spencer J, et al. Integrating human-centred design into the development of an intervention to improve the mental wellbeing of young women in the perinatal period: the Catalyst project. BMC Pregnancy Childbirth. 2021;21(1):183. doi: 10.1186/s12884-021-03675-y 33673826 PMC7936480

[56] Tirgari B, Rayyani M, Cheraghi MA, Mangeli M. Experiences of Iranian Teen Mothers with Parenting Stress: A Qualitative Study. Compr Child Adolesc Nurs. 2020;43(3):203–16. doi: 10.1080/24694193.2019.1651420 31412216

[57] Undie C-C, Birungi H. What to Expect When Girls are Expecting: Psychosocial Support Challenges and Opportunities in the Context and Aftermath of Teenage Pregnancy in Kenya. In Review; 2022 June. doi: 10.21203/rs.3.rs-1716775/v1 PMC9768923 36544207

[58] Wainaina CW, Sidze EM, Maina BW, Badillo-Amberg I, Anyango HO, Kathoka F, et al. Psychosocial challenges and individual strategies for coping with mental stress among pregnant and postpartum adolescents in Nairobi informal settlements: a qualitative investigation. BMC Pregnancy Childbirth. 2021;21(1):661. doi: 10.1186/s12884-021-04128-2 34583684 PMC8480022

[59] Webb L, Kyaddondo D, Ford T, Bergqvist A, Cox N. Psychosocial health in adolescent unmarried motherhood in rural Uganda: Implications for community-based collaborative mental health education, and empowerment strategies in the prevention of depression and suicide. Transcult Psychiatry. 2023;60(3):537–51. doi: 10.1177/13634615221147361 36628461 PMC10486171

[60] Yoosefi Lebni J, Khalajabadi Farahani F, Solhi M, Ebadi Fard Azar F. Causes and Grounds of Childbirth Fear and Coping Strategies Used by Kurdish Adolescent Pregnant Women in Iran: A Qualitative Study. J Reprod Infertil. 2021;22(1):47–56. doi: 10.18502/jri.v22i1.4995 33680885 PMC7903670

[61] Tinago CB, Frongillo EA, Warren AM, Chitiyo V, Jackson TN, Cifarelli AK, et al. Testing the Effectiveness of a Community-Based Peer Support Intervention to Mitigate Social Isolation and Stigma of Adolescent Motherhood in Zimbabwe. Matern Child Health J. 2024;28(4):657–66. doi: 10.1007/s10995-023-03821-2 37957412 PMC10963463

[62] Kassa GM, Arowojolu AO, Odukogbe ATA, Yalew AW. Adverse maternal outcomes of adolescent pregnancy in Northwest Ethiopia: A prospective cohort study. PLoS One. 2021;16(9):e0257485. doi: 10.1371/journal.pone.0257485 34550977 PMC8457495

[63] Yako EM. A comparative study of adoloscents’ perceived stress and health outcomes among adolescent mothers and their infants in Lesotho. Curationis. 2007;30(1):15–25. doi: 10.4102/curationis.v30i1.1035 17515312

[64] Sangsawang N, Sangsawang B. Postpartum depression, social support and maternal self-efficacy between adolescent and adult mothers during the COVID-19 pandemic: A comparative cross-sectional study. J Adv Nurs. 2023;79(1):113–24. doi: 10.1111/jan.15445 36117329

[65] Thomson KC, Romaniuk H, Greenwood CJ, Letcher P, Spry E, Macdonald JA, et al. Adolescent antecedents of maternal and paternal perinatal depression: a 36-year prospective cohort. Psychol Med. 2021;51(12):2126–33. doi: 10.1017/S0033291720000902 32340651

[66] Bennett HA, Einarson A, Taddio A, Koren G, Einarson TR. Prevalence of depression during pregnancy: systematic review. Obstet Gynecol. 2004;103(4):698–709. doi: 10.1097/01.AOG.0000116689.75396.5f 15051562

[67] Alvarado-Esquivel C, Sifuentes-Alvarez A, Salas-Martinez C. Depression in teenager pregnant women in a public hospital in a northern mexican city: prevalence and correlates. J Clin Med Res. 2015;7(7):525–33. doi: 10.14740/jocmr2156w 26015817 PMC4432894

[68] Wang Z, Liu J, Shuai H, Cai Z, Fu X, Liu Y, et al. Mapping global prevalence of depression among postpartum women. Transl Psychiatry. 2021;11(1):543. doi: 10.1038/s41398-021-01663-6 34671011 PMC8528847

[69] Musiimenta A. A Controlled Pre-Post Evaluation of a Computer-based HIV/AIDS Education on Students’ Sexual Behaviors, Knowledge and Attitudes. Online J Public Health Inform. 2012;4(1):ojphi.v4i1.4017. doi: 10.5210/ojphi.v4i1.4017 23569630 PMC3615807

[70] Anderson C, Logan D. Impact of traumatic birth experience on Latina adolescent mothers. Issues Ment Health Nurs. 2010;31(11):700–7. doi: 10.3109/01612840.2010.518784 20936891

[71] Lowe NK. Self-efficacy for labor and childbirth fears in nulliparous pregnant women. J Psychosom Obstet Gynaecol. 2000;21(4):219–24. doi: 10.3109/01674820009085591 11191169

[72] Al-Kloub MI, Al-Zein HJ, Abdalrahim MS, Abed MA. Young women’s experience of adolescent marriage and motherhood in Jordan. Cult Health Sex. 2019;21(4):462–77. doi: 10.1080/13691058.2018.1489067 30355056

[73] Kotzé A. The experience of early motherhood amongst Swazi Adolescent Girls. North-West University. 2014. Available from: http://hdl.handle.net/10394/15339

[74] Murry VM, Heflinger CA, Suiter SV, Brody GH. Examining perceptions about mental health care and help-seeking among rural African American families of adolescents. J Youth Adolesc. 2011;40(9):1118–31. doi: 10.1007/s10964-010-9627-1 21259067

[75] Halbreich U, Karkun S. Cross-cultural and social diversity of prevalence of postpartum depression and depressive symptoms. J Affect Disord. 2006;91(2–3):97–111. doi: 10.1016/j.jad.2005.12.051 16466664

[76] Klainin P, Arthur DG. Postpartum depression in Asian cultures: a literature review. Int J Nurs Stud. 2009;46(10):1355–73. doi: 10.1016/j.ijnurstu.2009.02.012 19327773

[77] Mitchell SJ, Lewin A, Horn IB, Valentine D, Sanders-Phillips K, Joseph JG. How does violence exposure affect the psychological health and parenting of young African-American mothers?. Soc Sci Med. 2010;70(4):526–33. doi: 10.1016/j.socscimed.2009.10.048 19932932 PMC2853478

[78] Fisher J, Cabral de Mello M, Patel V, Rahman A, Tran T, Holton S, et al. Prevalence and determinants of common perinatal mental disorders in women in low- and lower-middle-income countries: a systematic review. Bull World Health Organ. 2012;90(2):139G-149G. doi: 10.2471/BLT.11.091850 22423165 PMC3302553

[79] Kapungu C, Petroni S, Allen NB, Brumana L, Collins PY, De Silva M, et al. Gendered influences on adolescent mental health in low-income and middle-income countries: recommendations from an expert convening. Lancet Child Adolesc Health. 2018;2(2):85–6. doi: 10.1016/S2352-4642(17)30152-9 30169241

[80] Prost A, Lakshminarayana R, Nair N, Tripathy P, Copas A, Mahapatra R, et al. Predictors of maternal psychological distress in rural India: a cross-sectional community-based study. J Affect Disord. 2012;138(3):277–86. doi: 10.1016/j.jad.2012.01.029 22342117 PMC3343258

[81] Shidhaye R, Patel V. Association of socio-economic, gender and health factors with common mental disorders in women: a population-based study of 5703 married rural women in India. Int J Epidemiol. 2010;39(6):1510–21. doi: 10.1093/ije/dyq179 21037247 PMC2992631

[82] Upadhyay RP, Chowdhury R, Aslyeh Salehi, Sarkar K, Singh SK, Sinha B, et al. Postpartum depression in India: a systematic review and meta-analysis. Bull World Health Organ. 2017;95(10):706-717C. doi: 10.2471/BLT.17.192237 29147043 PMC5689195

[83] Roberts KJ, Smith C, Cluver L, Toska E, Zhou S, Boyes M, et al. Adolescent Motherhood and HIV in South Africa: Examining Prevalence of Common Mental Disorder. AIDS Behav. 2022;26(4):1197–210. doi: 10.1007/s10461-021-03474-8 34570313 PMC8940800

[84] Arenson JD. Strengths and self-perceptions of parenting in adolescent mothers. J Pediatr Nurs. 1994;9(4):251–7. 7965593

[85] SmithBattle L, Leonard VW. Adolescent mothers four years later: narratives of the self and visions of the future. ANS Adv Nurs Sci. 1998;20(3):36–49. doi: 10.1097/00012272-199803000-00006 9504207

[86] De La Rey C, Parekh A. Community-based Peer Groups: an Intervention Programme for Teenage Mothers. J Community Appl Soc Psychol. 1996;6(5):373–81. doi: 10.1002/(sici)1099-1298(199612)6:5<373::aid-casp388>3.0.co;2-o

[87] Sanders MR, Divan G, Singhal M, Turner KMT, Velleman R, Michelson D, et al. Scaling Up Parenting Interventions is Critical for Attaining the Sustainable Development Goals. Child Psychiatry Hum Dev. 2022;53(5):941–52. doi: 10.1007/s10578-021-01171-0 33948778 PMC8096135

[88] Kuipers YJ, Beeck E van, Cijsouw A, van Gils Y. The impact of motherhood on the course of women’s psychological wellbeing. J Affect Disord Rep. 2021;6:100216. doi: 10.1016/j.jadr.2021.100216

[89] Poudel S, Razee H, Dobbins T, Akombi-Inyang B. Adolescent Pregnancy in South Asia: A Systematic Review of Observational Studies. Int J Environ Res Public Health. 2022;19(22):15004. doi: 10.3390/ijerph192215004 36429723 PMC9690629

[90] Patel V, Saxena S, Lund C, Thornicroft G, Baingana F, Bolton P, et al. The Lancet Commission on global mental health and sustainable development. Lancet. 2018;392(10157):1553–98. doi: 10.1016/S0140-6736(18)31612-X 30314863

[91] Ali A, Khaliq A, Lokeesan L, Meherali S, Lassi ZS. Prevalence and predictors of teenage pregnancy in Pakistan: a trend analysis from Pakistan Demographic and Health Survey datasets from 1990 to 2018. Int Health. 2022;14(2):176–82. doi: 10.1093/inthealth/ihab025 34013327 PMC8890806

[92] Mehra R, Alspaugh A, Dunn JT, Franck LS, McLemore MR, Keene DE, et al. “‘Oh gosh, why go?’ cause they are going to look at me and not hire”: intersectional experiences of black women navigating employment during pregnancy and parenting. BMC Pregnancy Childbirth. 2023;23(1):17. doi: 10.1186/s12884-022-05268-9 36627577 PMC9830615

[93] Carastathis A. The Concept of Intersectionality in Feminist Theory. Philos Compass. 2014;9(5):304–14. doi: 10.1111/phc3.12129

[94] Meleis AF. Transitions theory: Middle range and situation specific theories in nursing research and practice. Springer Pub; 2010.

[95] Hutchinson AJ. Surviving, Coping or Thriving? Understanding Coping and Its Impact on Social Well-Being in Mozambique. Br J Soc Work. 2012;44(4):972–91. doi: 10.1093/bjsw/bcs167

