# Supplementary material for: Motherhood and mental health of adolescent girls in low- and middle-income countries: A scoping review
Source: PLOS Glob Public Health. 2025 Sep 17;5(9):e0005134. doi: 10.1371/journal.pgph.0005134 (PMC12443265; doi:10.1371/journal.pgph.0005134)
Supplement: S1 File — (DOCX) [file pgph.0005134.s002.docx]

**S1 File**

**Search strategy for CINAHL**


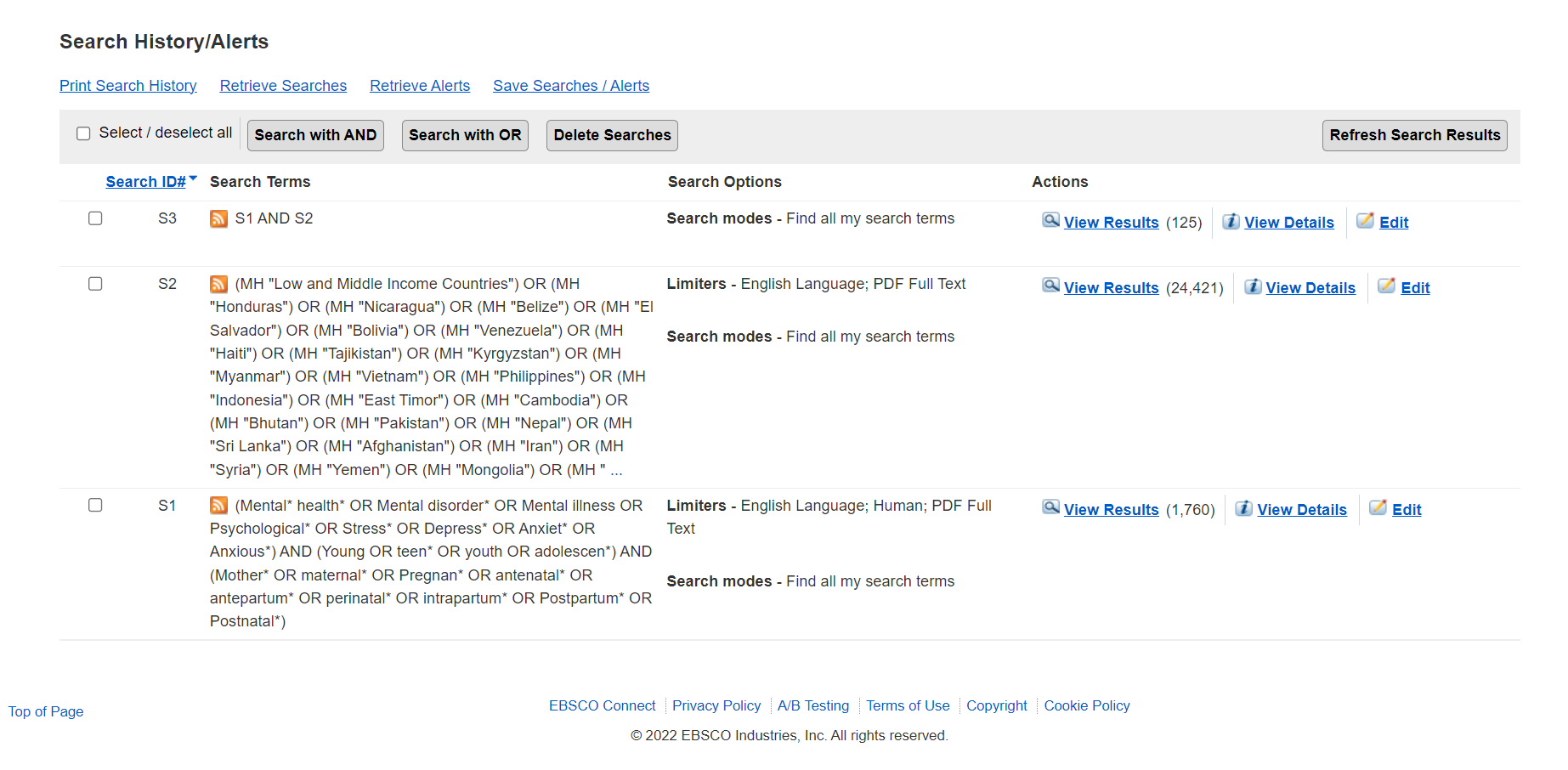


**Search strategy for ERIC**

**
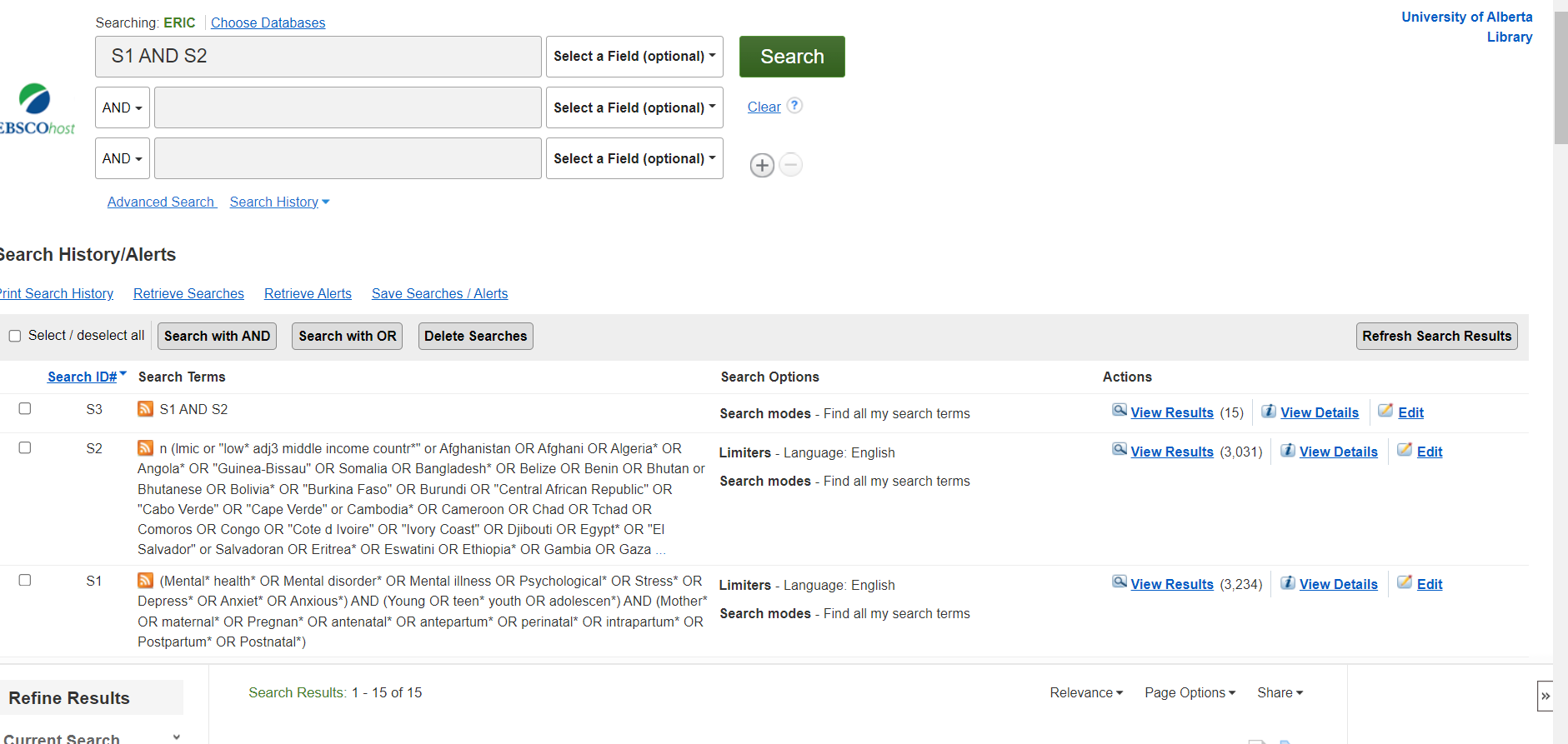
**

**Search strategy for MEDLINE**


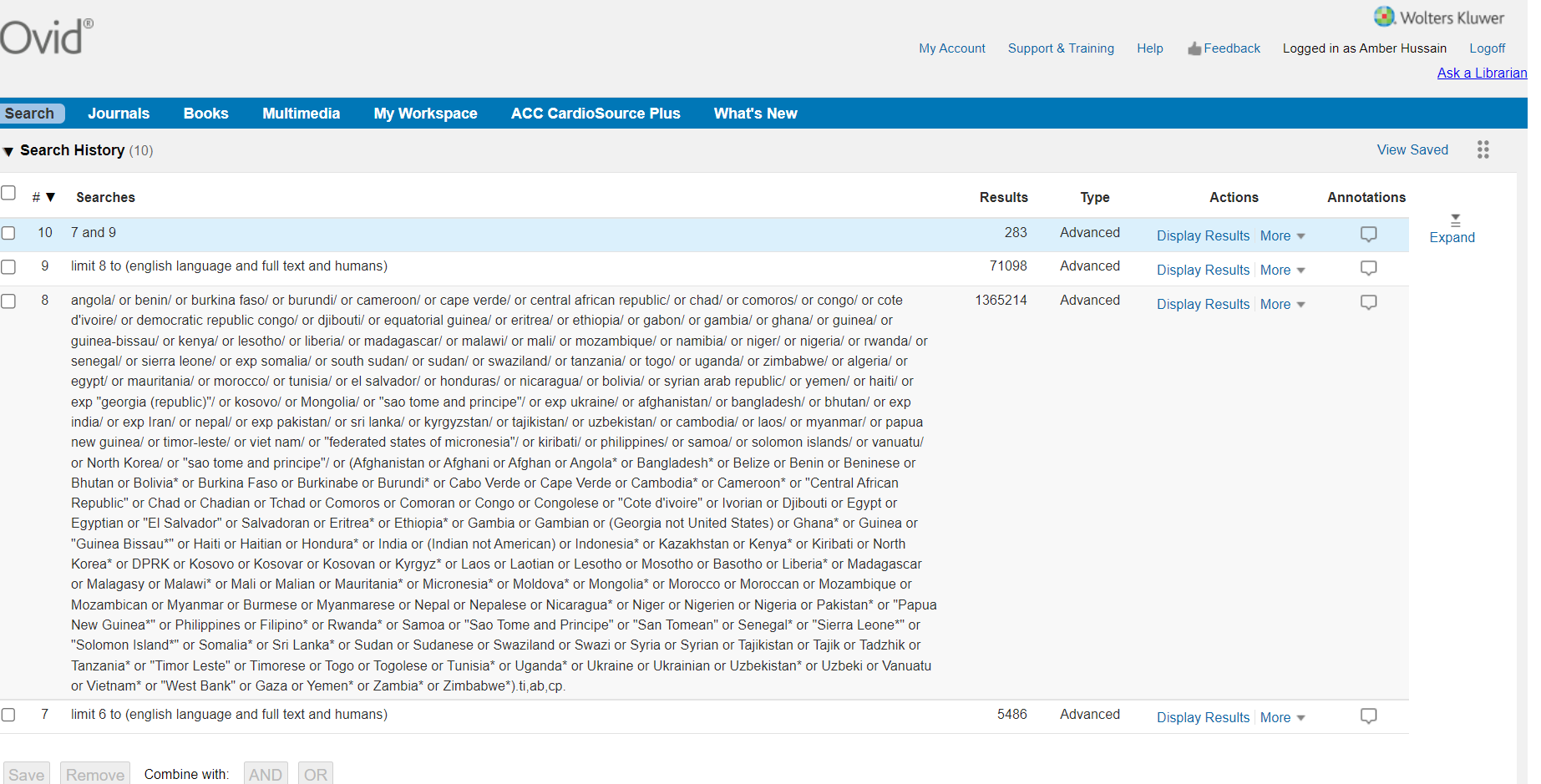


**Search strategy for PsycINFO**

**
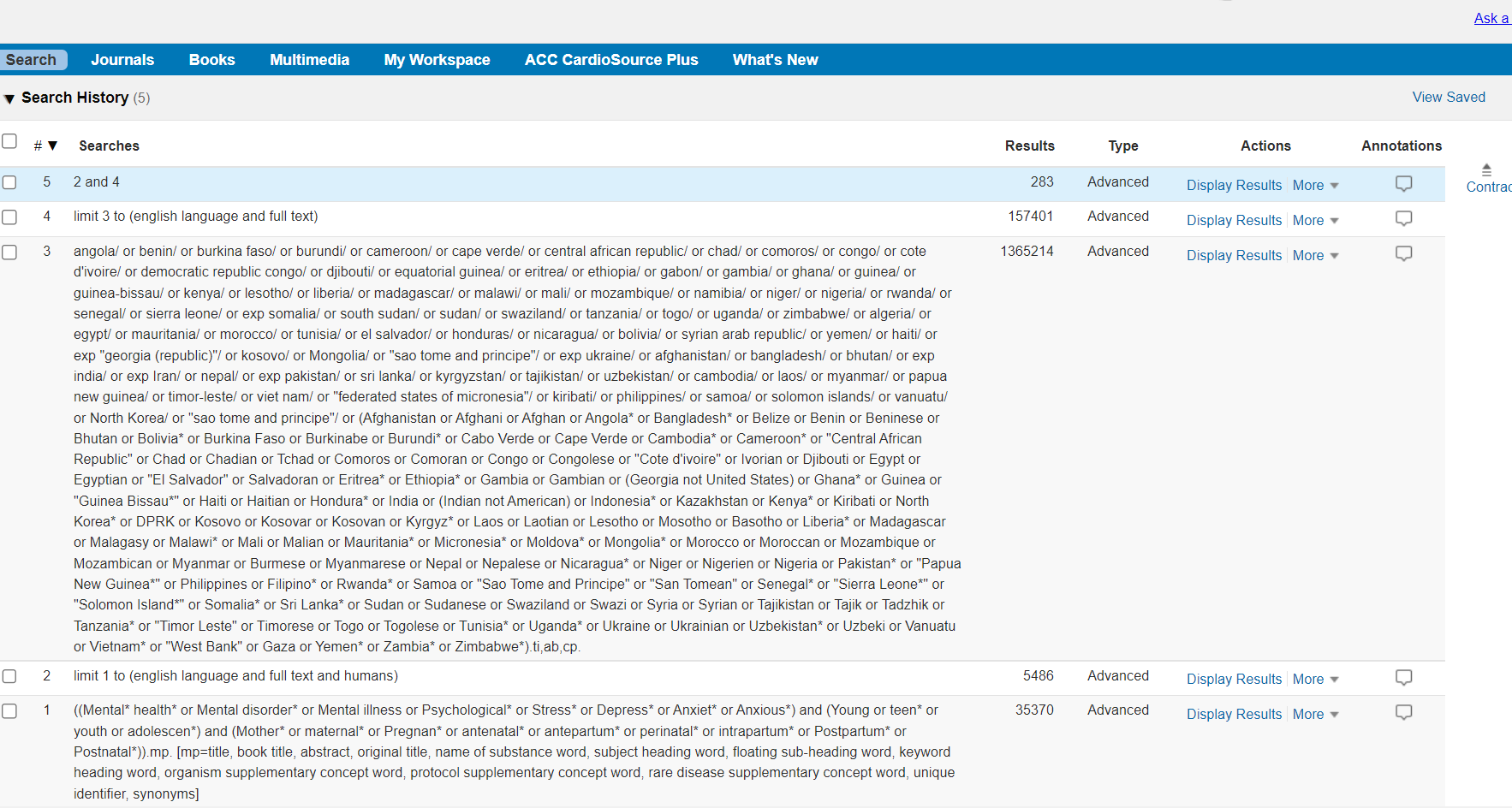
**

**Search strategy for EMBASE**

**
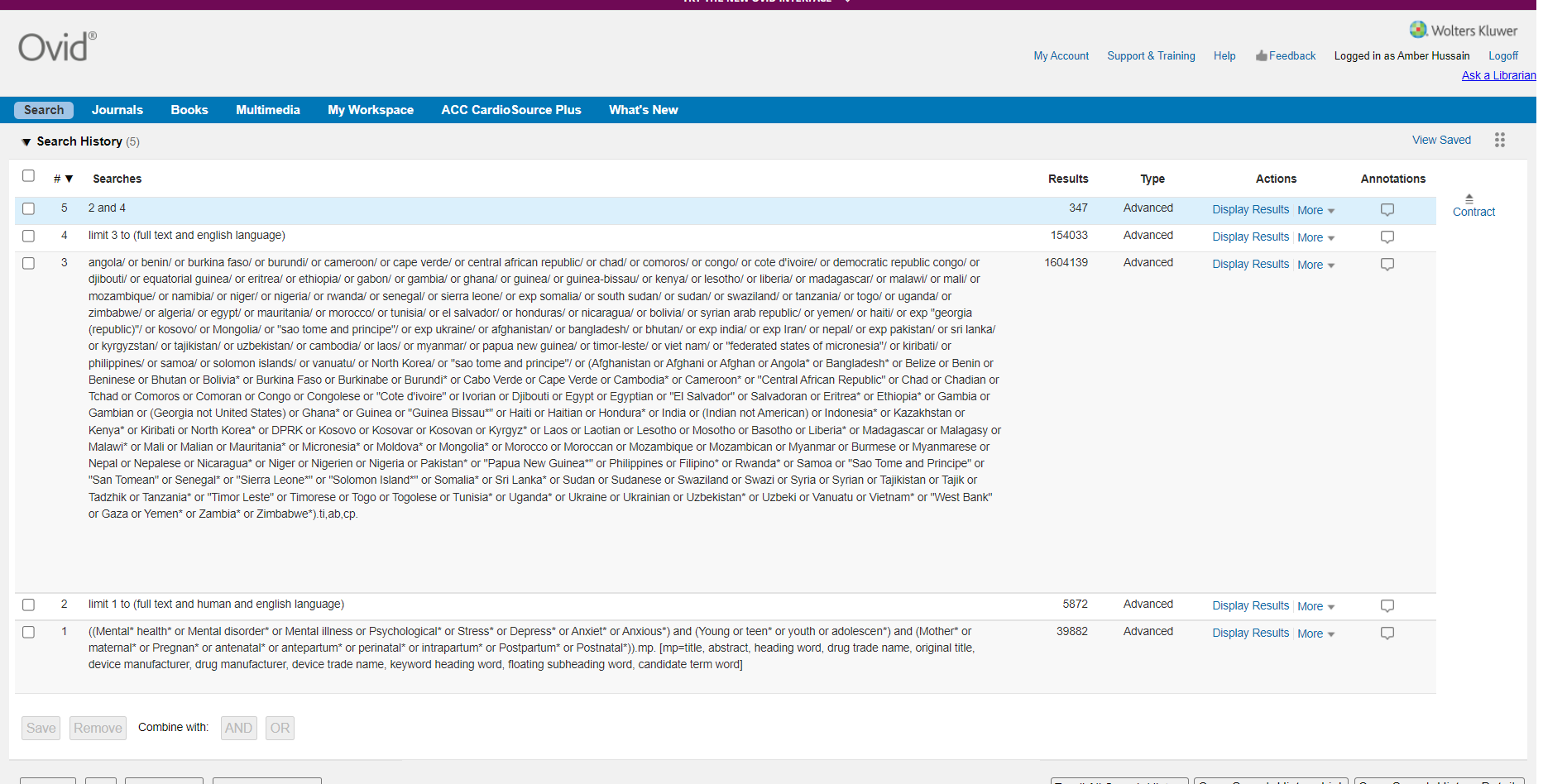
**

**Search strategy for Global Health**

**
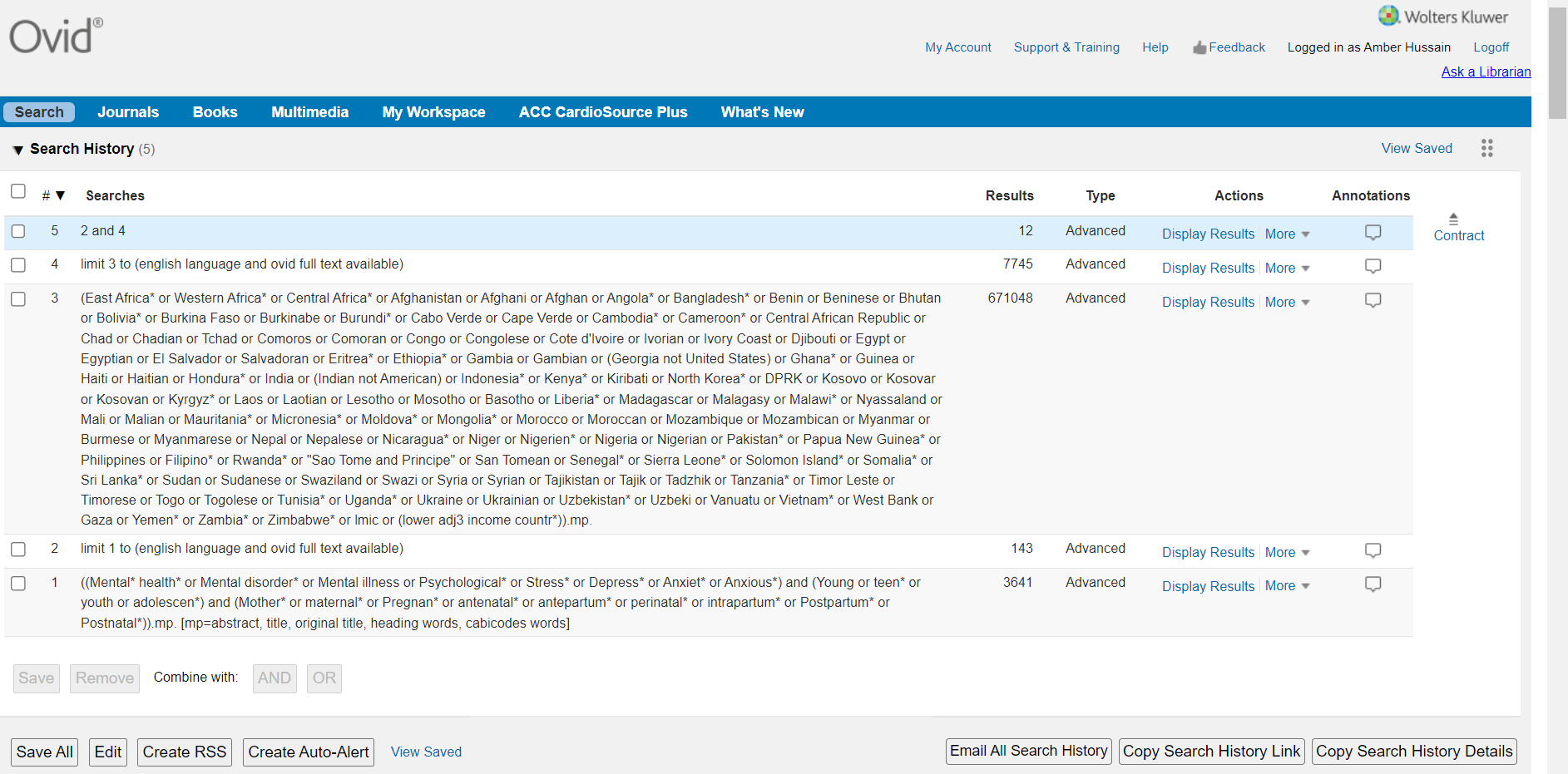
**

**Search strategy for ProQuest**

**
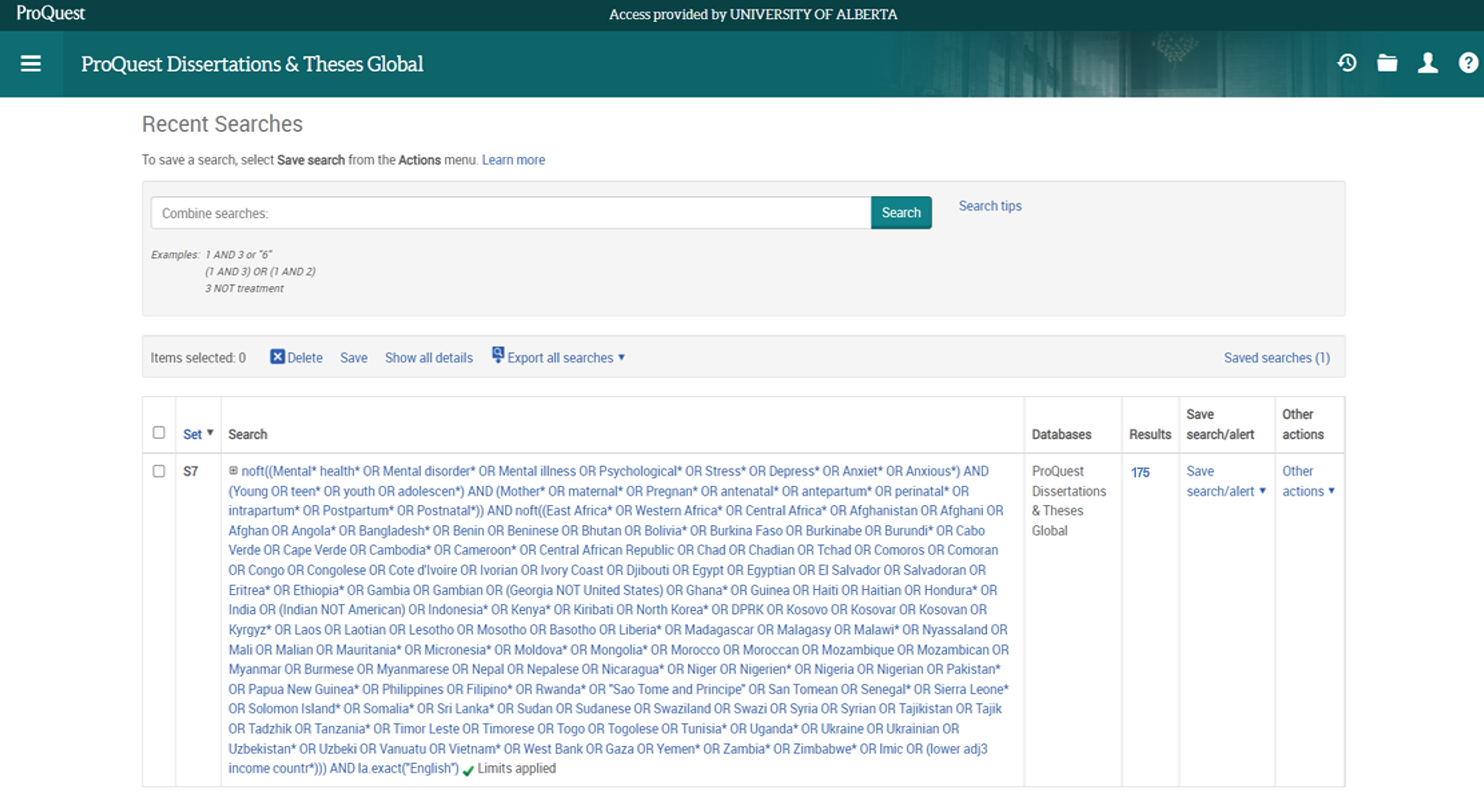
**
