# Supplementary material for: Motherhood and mental health of adolescent girls in low- and middle-income countries: A scoping review
Source: PLOS Glob Public Health. 2025 Sep 17;5(9):e0005134. doi: 10.1371/journal.pgph.0005134 (PMC12443265; doi:10.1371/journal.pgph.0005134)
Supplement: S2 File — (DOCX) [file pgph.0005134.s003.docx]

**S2 File. Data extraction table**

| **Authors and Year** | **Country, Region and Income Level** | **Aim of study** | **Theoretical Framework** | **Study Design, Method, sampling techniques** | **Target Population, Age & Sample size** | **Main Findings** |
| --- | --- | --- | --- | --- | --- | --- |
| Agampodi et al., 2021 | Sri Lanka, Rural (Anuradhapura)  Income level: Lower middle income  Region: South Asia | To describe the hidden burden, associated biological and psychosocial factors and utilization patterns of pre-conceptional services among pregnant adolescents in rural Sri Lanka. | None | Quantitative  prospective cohort  Study, self-administered questionnaire (Edinburgh Postpartum Depression  Scale)  Sampling technique not mentioned | Pregnant adolescent women (25–28 weeks of gestation), 15-19 years  n=233 | Adolescent mothers were less happy of being pregnant (p = 0.006) and had significantly higher levels of anxiety (p = 0.009). Depression (p=0.461) and anhedonia (p=0.416) |
| Ajayi et al., 2023 | Burkina Faso  and Malawi  Urban and rural areas  Income level: Both Low income  Region: Sub-Saharan Africa | To examine the socio-ecological factors associated with depression symptoms among pregnant and parenting adolescent girls. | Socioecological model | Quantitative  Cross-sectional  Global Early Adolescent Study tools, and Patient Health Questionnaire-9 (PHQ-9)  Two-stage probability sampling (Random and household listing) | Pregnant and parenting girls,  10-19 years  n=669 | Probable depression rates were 18.8% in Burkina Faso and 14.5% in Malawi. In Malawi, secondary education correlated with lower depression likelihood (AOR: 0.47), while in Burkina Faso, it didn't. Family factors, such as denying paternity and lack of parental support, increased depression odds. |
| Belete et al., 2021 | Ethiopia Public Hospital  Income level: Low income  Region: Sub-Saharan Africa | To assess the prevalence and factors associated with suicide ideation and attempt among pregnant women attending antenatal care services at public hospitals in  southern Ethiopia. | None | Quantitative  Cross-sectional  Composite International Diagnostic Interview (CIDI) by WHO, SRQ-20 by WHO, and WHO violence measure  Systematic random sampling | Pregnant women,  15-49 years  Total participants=762  Adolescent sample (15-19 years) n= 28 | For 15-19 years:4 (14.3) had current suicidal ideation, COR=1.78 (0.53-5.98), AOR=1.23 (0.25-6.03) |
| Kassa et al, 2021 | Ethiopia Seven districts in the East Gojjam zone, Northwest.  Income level: Low income  Region: Sub-Saharan Africa | To assess the adverse maternal outcomes of adolescent pregnancy in Northwest Ethiopia | None | Quantitative, Prospective Cohort  Edinburgh Postnatal Depression Scale (EPDS)  Multistage sampling | Adolescent and adult women adolescents’ age  15-19 years  Total participants=1,134  Adolescent sample (15-19 years): n= 374 | A considerably larger proportion of teenage women experienced PPD (37.4%) compared to adult women (20.1%), p-value <0.000 |
| Kaye, 2008 | Kampala, Uganda  Mulago hospital  Income level: Low income  Region: Sub-Saharan Africa | explore what adolescents perceived as their struggles during the period of transition from childhood to parenthood and specifically, describe strategies employed in coping with stress of pregnancy, motherhood and parenthood. | Stress and coping model | Qualitative Longitudinal study  In-depth interviews and focus group discussions.  Theoretical sampling | Pregnant adolescents followed from pregnancy to delivery, 14-19 years.  n=52 | Adolescents reported anxiety, loss of self-esteem, difficulty in accessing financial, moral and material support from parents or partners and stigmatization by health workers when they sought care from health facilities. Three strategies by  which adolescent mothers cope with parenting and pregnancy stress that were described as utilizing opportunities, accommodating the challenges, or failure, and varied in the extent to which they enabled adolescents to cope with the stress. |
| Khanna, 2021 | India  Rural  Income level: Lower middle income  Region: South Asia | To determine whether gender disadvantage  factors are associated with psychological distress among young women in rural India. | None | Quantitative  Cross-sectional  GHQ-12 item questionnaire.  Systematic random  sampling | Young married women, 15-24 years  n=229 | Psychological distress was found among 21.9%. Young women who were married before 18 years had 2.19 times higher odds of distress than women who were married after 18 years. Young women who gave birth to a female infant had 2.43 times higher odds of distress than those who gave birth to a male infant. Lack  of partner support and experience of postnatal health  complications were other predictors. |
| Kimbui et al., 2018 | Kenya Nairobi, Health Centre  Income level: Lower middle income  Region: Sub-Saharan Africa | To identify social determinants of mental health such as social support, partner or parent support, and demographic profile | None | Quantitative  cross-sectional  Edinburgh  Postnatal Depression Screen questionnaire  Purposive Sampling | Pregnant adolescents,  16-18 years  n=212 | 43.1% had depression, among which 60.4% had depressive symptoms, and 51.9% had severe depression scores. 26.9% were currently consuming alcohol. The more severely depressed participants had greater alcohol use. Of the 110 pregnant adolescents who were severely depressed, 39 were currently consuming alcohol. Alcohol use disorder factors associated with depression included living with an alcoholic, ever and current use of alcohol, alcohol-related harm being experienced, being pressured to take alcohol |
| Klingberg-Allvin et al., 2008 | Vietnam Rural district  Income level: Lower middle income  Region: East Asia & Pacific | To explore adolescents’ perceptions and experiences related to transition into motherhood and their encounter with health care service. | None | Qualitative  In-depth semi structured interviews  The sampling technique not mentioned | Pregnant or newly delivered adolescent, less than 20 years  n=22 | young women experienced ambivalence in the transition to motherhood in that they felt too young but also happy to be able to please their husband and the extended family. However, participants experienced lacking power with regard to decisions in relation to pregnancy, delivery, and contraceptive usage. They also had feelings of being patronized and ignored in the encounter with health care providers. |
| Kola et al., 2020 | Nigeria  Income level: Lower middle income  Region: Sub-Saharan Africa | To identify factors influencing  health service utilization for adolescent perinatal depression, in Nigeria to inform new strategies of care delivery. | Behavioral Model | Qualitative  Focus group.  Purposive sampling | Adolescent mothers with perinatal depression, age not mentioned.  Also included providers  n=17 mothers and 25 providers | Perceived health benefits of treatment  received for perinatal depression were strong motivation for service use. The service environment was often discouraging, with long queues and stigmatizing attitude by many of the providers. In the clinic, the stigma was about both their early pregnancy and their depression. |
| Labrague et al., 2020 | Philippines Rural area  Income level: Lower middle income  Region: East Asia & Pacific | To explore the prevalence and predictors of postpartum depression (PPD) as well as the utilization and evaluation of PPD services among postpartum women in rural areas of the Philippines. | None | Quantitative  Cross-sectional study  Edinburgh Postnatal Depression Scale (EPDS)  Convenience Sampling | Women who visited maternal facilities (no age limit)  Total sample =165, Adolescent less than 19 years n=39 | Age <19 years (N=39): 9 (23.1%) had PPD |
| Li et al., 2021 | Bangladesh  Urban & Rural areas  Income level: Lower middle income  Region: South Asia | To assess the prevalence of suicide attempts  among young women with adolescent pregnancy in Bangladesh and to explore its associated factors | None | Quantitative  Cross-sectional study  Suicide-Screening  Questions Toolkit  Sampling not mentioned | Pregnant women,  Less than 18 years  n=940 | 6.5% reported suicide attempts in the past 12 months, and the majority (88.5%) of the attempts happened within one year after the pregnancy. Participants with more years after first pregnancy and more perceived social support from friends were less likely to have suicide attempts, and those perceived bad health status compared with good/fair health status were more likely to attempt suicide. |
| Mark et al., 2021 | Malawi Rural Ntcheu District  Income level: Low income    Region: Sub-Saharan Africa | To evaluate the association between food insecurity (FI) and clinical depression and the modifying effects of seasonality on this association | None | Quantitative,  Cross-sectional  Food insecurity: USAIDs Household Food Insecurity Access Scale (HFIAS). PPD: Chichewa version of the Self-Reporting Questionnaire (SRQ).  The sampling technique not mentioned | Postpartum women (till 6 months of delivery) 16 or older  Total participants=175  Adolescent sample (16-19 years): n=39 | Adolescent (16-19):5 (14.7%) had clinical postpartum depression.  Crude and adjusted odds of meeting clinical  depression predicted by food insecurity among adolescents (16-19) vs. young (20-29): OR=0.898 (0.263-3.063) p=0.25 |
| Musyimi et al., 2020 | Kenya  Income level: Lower middle income  Region: Sub-Saharan Africa | To investigate the factors  associated with suicidal behavior among adolescent pregnant mothers in Kenya. | Socio-ecological model | Qualitative  Interviews and focus group interviews.  Purposive and snowball sampling | adolescent mothers, 15–19 years)  n=21  8 Key Informant Interviews were also interviewed | Poverty, intimate partner violence (IPV), family rejection, social isolation and stigma from the community, and chronic physical illnesses were associated with suicidal ideations. |
| Nasreen et al., 2011 | Bangladesh Rural  Income level: Lower middle income  Region: South Asia | To estimate the prevalence of depressive and anxiety symptoms and explore the associated factors in a cross-section of rural Bangladeshi pregnant women. | None | Quantitative  Cross-sectional data  Edinburgh Postnatal Depression Scale (EPDS)  Random Sampling | Women in their third trimester of pregnancy  Total participants=720  Adolescent sample (less than 19 years): n= 158 | 16.7% adolescent had antepartum depressive symptoms |
| Nicolet et al., 2021 | Cameroon  Urban  Income level: Lower middle income  Region: Sub-Saharan Africa | To identify and explore factors of perinatal depression among teenage mothers in Cameroon. | None | Quantitative  Edinburgh Postnatal Depression Scale  Sampling strategy not mentioned | Women in perinatal period, less than 20 years  n=1344 | The prevalence of depressive disorder symptoms among teenage or young pregnant women is estimated to be 70.0%. This risk is significantly increased by different factors including unintended or unplanned pregnancy, experiencing depression and anxiety before childbirth, and domestic violence. |
| Nurbaeti et al., 2023 | Indonesia  Income level: Lower middle income  Region: East Asia Pacific | To address the mental health and well-being of postpartum mothers in Indonesia, especially among adolescents | None | Quantitative  Cross-sectional  The Edinburgh Postnatal Depression Scale  Cluster sampling | Muslim adolescent mothers in the postpartum period ranging, less than 20 years.  n=203 | 35.96% of teenage mothers experienced symptoms of postpartum depression. Marriage satisfaction, education level, family income, number of children, and baby weight at birth were significantly associated with postpartum depression. However, social support and religiosity showed no significant association with postpartum depression. |
| Okine et al., 2020 | Ghana Mamprobi Polyclinic, Accra  Income level: Lower middle income  Region: Sub-Saharan Africa | To explore challenges faced by teenage mothers with repeat pregnancy and the support services available to them. | Theory/Framework: Bronfenbrenner’s (1979) ecological systems theory | Qualitative  In-depth interviews &focus group discussions  Convenience and Snowball sampling | Teenage mothers, less than 20 years  Also included Health care workers  n=33 teenage and 8 HCW | Challenges include educational, financial, and health challenges. Adolescents experienced psychological issues, particularly stigma. They had feeling feelings of shame, anger and rejection, depression, and suicidal ideations. stigma was an issue they had to deal with daily. |
| Okunola et al., 2022 | Nigeria semi-urban community Sampling technique not mentioned.  Income level: Lower middle income  Region: Sub-Saharan Africa | To evaluate the relationship between antenatal depression (APD) and postpartum depression (PPD) and predictors of PPD among an obstetric population in South-Western Nigeria. | None | Quantitative  a prospective longitudinal cohort study  Edinburgh Postnatal Depression Scale (EPDS)  Sampling strategy not mentioned | Pregnant women between 34-36 weeks (follow-up up till 6 weeks of delivery)  Total participants=272  Adolescent sample (15-24 years): n= 29 | 15-24 years: 27.6% were depressed |
| Osok, Kigamwa, Huang, et al., 2018 | Nairobi Kenya Health facility antenatal service  Income level: Lower middle income  Region: Sub-Saharan Africa | To elicit various practical, psychological, interpersonal, and cultural barriers to life adjustment, service access, obtaining resources, and psychosocial support related to pregnancy. | None | Qualitative Grounded theory  In-depth semi-structured interviews (engagement interview approach)  Purposive sampling | first-time Pregnant adolescents,  15-19 years  n=12 | Challenges, including depression, anxiety and stress around the pregnancy, denial of the pregnancy, lack of basic needs provisions and care, and restricted educational or livelihood opportunities for personal development post pregnancy. These challenges led to negative mental health consequences in adolescent pregnant girls, including feeling insecure about the future, feeling very defeated and sad to be pregnant, and feeling unsupported and disempowered in providing care for the baby. |
| Osok, Kigamwa, Stoep, et al., 2018 | Nairobi, Kenya urban resource-deprived areas  Income level: Lower middle income  Region: Sub-Saharan Africa | To determine the prevalence of depression and related psychosocial risks among pregnant adolescents reporting at a maternal and child health clinic in Nairobi, Kenya. | None | Quantitative Cross-sectional  HQ-9 to assess depression.  Convenient sampling | Pregnant adolescents, 15-18 years  n=176 | 2.9% (n = 58) had antenatal depression. Predictors include stressful life events, caregiver burden, absence of social support, being diagnosed with HIV/AIDS and being young. |
| Puey, 2022 | Philippines colleges/University  Income level: Lower middle income  Region: East Asia & Pacific | To explore young mothers' lived experiences and challenges at the University of Southern Mindanao. | None | Qualitative phenomenology  Structured In-depth Interview  Purposive sampling | Teenage mothers, 24 years or younger  n=25 | Adolescent had psychological challenges including felt rejected, embarrassed and had broken relationship. |
| Putri., 2023 | Indonesia  Urban and rural  Income level: Lower middle income  Region: East Asia Pacific | To assess postpartum depression symptoms in young mother | None | Quantitative  Cross-sectional survey  Mini International Neuropsychiatric Interview (MINI) instrument  Systematic linear sampling | Young mothers with babies less than 6 weeks, 15-24 years  n=1,285 | The prevalence of postpartum depression in the 6 months postpartum was 4.0%, with a higher prevalence in urban areas (5.7%) than in rural areas (2.9%). Post partum depression was associated with living without a husband, experiencing preterm birth, having pregnancy complications, unwanted pregnancy, and having postpartum complications were associated with a higher risk of postpartum depression. |
| Taylor Salisbury et al., 2021 | Mozambique, in the District of Manhiça  Income level: Low income  Region: Sub-Saharan Africa | To understand the experiences, causes, and priorities challenges that affect the mental health of young Mozambican mothers during the perinatal  Period, and to co-design possible interventions | None | Qualitative  Human centered design approach  Focus-group discussions, individual interviews, and observations.  Sampling technique not mentioned | Women who were pregnant or given birth within a year,  16-24 years  n=23 women  Also included 12 family members, 19 service providers and 11 staff from the Ministry of Health. | Uncertainty focused on living situations, pregnancy outcomes, parenting, education, and financial stability, social support and limited knowledge. Several (39 %) were happy about their pregnancy and felt emotionally and materially supported. Those with unplanned pregnancies, had mixed or negative feelings towards the pregnancy and the impact. |
| Tele et al., 2022 | Nairobi Kenya  Income level: Lower middle income  Region: Sub-Saharan Africa | To find prevalence of depression and its  associated risk factors among pregnant adolescents in Nairobi, Kenya | None | Quantitative cross-sectional  Patient Health Questionnaire 9  Purposive sampling | pregnant adolescent, 14-18 years  n=153 | 43.1% of the respondents were depressed.  Depressive symptoms in were independently associated with being in school, experience of intimate partner violence, substance use within the family and having experienced pressure to use substances by family or peers. |
| Tembo et al., 2023 | Malawi Rural  Income level: Low income    Region: Sub-Saharan Africa | To identify the prevalence and social and cultural influences of depression among adolescents  mothers attending community clinics at Mitundu in Lilongwe, Malawi. | None | Quantitative Cross-sectional  Edinburgh Postnatal  Depression Scale  Convenience sampling | Adolescent postnatal mothers. Less than 19 years  n=395 | 43.6% (n = 172) presented with Postnatal depression (PND). Adolescents who had ever experienced intimate partner violence (IPV) were 13.6 times more likely to report PND, Participants whose families did not decide for them (regarding their care) were 2.3 times more likely to present with PND than those whose families, adolescent mothers who had interacted with their health worker were less likely to report PND than those who had no interaction with the health worker. |
| Tinago et al., 2023 | Zimbabwe  Income level: Lower middle income  Region: Sub-Saharan Africa | To test the effectiveness  of a community-based peer support intervention to mitigate social isolation and stigma of adolescent motherhood  in Harare, Zimbabwe | None | Quasi experimental  Patient Health Questionnaire  (PHQ-9)  Purposive and Snowball sampling | Adolescent mothers, 14–18 years  n=183 (intervention group=104 and control group=79) | The intervention arm reported lower depressive symptoms and common mental disorders and higher overall, family, friends, and significant-other support, compared to control. The intervention arm felt more engaged with peers, knew who and where to turn to for help, and had coping, parenting and communication strategies to manage life challenges. |
| Tirgari et al., 2020 | Iran (Kerman province) Health centres  Income level: Lower middle income  Region: Middle East & North Africa | To explore experiences of teen mothers with stress and stressors of early motherhood | None | Qualitative  In-depth semi-structured interviews  Purposive sampling | Teen mothers less than 19 years  n=18 | Five categories: 1) storm of anxiety (s fear and worry, regret and helplessness, guilty and ashamed, depression, loneliness, and isolation) 2) wander identity (Conflict between maternal and adolescence roles Role strain) 3) an unaccompanied way, (Insufficient familial and health support) 4) unarmed combat, (Knowledge and skill insufficiency) 5) and a tired body (Increasing responsibilities related to motherhood) |
| Undie & Birungi, 2022 | Kenya  Income level: Lower middle income  Region: Sub-Saharan Africa | To examine the experience of teenage pregnancy and the resultant psychosocial support needs from the perspectives of both pregnant/parenting girls and their own parents, who are  typically expected to provide various forms of support. | None | Qualitative  Descriptive case study design  Secondary data from counselling notes  Sampling strategy not mentioned | Pregnant or parenting girls, teenage  n=20 | Pregnant/parenting girls showed evidence of psychological trauma as a result of their pregnancies. The sexual debut of many girls occurred in the context of sexual violence wide range of psychological problems and mental health issues described by pregnant and parenting girls, including depression, fear, suicidal ideation, insomnia, reduced self-esteem, and hopelessness, |
| Uzobo, 2022 | Nigeria  Bayelsa State (in the health centre and clinic)  Income level: Lower middle income  Region: Sub-Saharan Africa | To examine the prevalence and coping strategies of Postnatal Depression (PND) among mothers in Bayelsa, Nigeria. | None | Quantitative  Cross-sectional  Self-developed questionnaire and Edinburgh’s scale  Purposive sampling | Women with babies between 1-6 weeks, 15-44 years  Total participants=345  Adolescent sample (15-19 years): n= 21 | 15-19 years: Of all mothers, 21 (16.2%) had mild depression Findings of coping strategies were not reported separately. |
| Vahidi et al., 2023 | Iran  Urban and sub-urban  Income level: Lower middle income  Region: Middle East & North Africa | To investigate relationship between childbirth experience and PTSD with maternal functioning in Iranian adolescent mothers. | None | Quantitative Cross-sectional  PTSD Symptom Scale, Childbirth  Experience Questionnaire 2.0, and Barkin Index of Maternal Functioning  Census Sampling | Adolescent women with one month and a maximum of 3 months have passed since giving birth,  10-19 years  n=202 | There was a statistically significant relationship between PTSD, childbirth experience and unwanted baby sex with maternal functioning. |
| Wainaina et al., 2021 | Nairobi, Kenya  Income level: Lower middle income  Region: Sub-Saharan Africa | To generate an  inventory of mental stressors during pregnancy and early motherhood; understand how mental stress affects the ability to seek care for themselves and their child and understand individual coping strategies. | None | Qualitative  Interviews and discussions from visual methodologies including Photovoice, digital storytelling,  and public service announcements  Sampling strategy not mentioned. | Pregnant and adolescent mothers, 14–19 years  n=30 | The psychosocial challenges identified in order of importance included: chased from home by the parents; economic hardship; neglect and abandonment by the person responsible for the pregnancy; stigmatization by family, friends, and the community; feelings of shattered dreams; and daily stress related to living in poor and unhygienic conditions. During the pregnancy and early motherhood, the participants experienced feelings of embarrassment, shame, hopelessness, and to the extreme, suicidal thoughts clouded their minds. Main coping strategies included social isolation for some, socializing with other pregnant and adolescent mothers, and negative behaviors like the uptake of illicit drugs and alcohol and risky sexual relationships. |
| Webb et al., 2023 | Uganda  Income level: Low income  Region: Sub-Saharan Africa | To explore the particular mental health impact of unmarried pregnancy among young rural Ugandan girls. | None | Qualitative ethnographic approach  Fieldwork, group meetings, visual artifacts, and written records  Sampling strategy not mentioned | Teenage mothers,  15-21 years  n=47 | Mental health symptoms include isolation and social withdrawal, disruptive or disorganised behaviour, overthinking, substance use, sense of guilt or self-blame, anger and strong emotions, helplessness, grief. Poverty, gender inequality, patriarchy, poor family support, Community and family views (stigma and shame) were found to be contributors. Barriers to service utilization include attitudes, traditional/religious beliefs and poor infrastructure. |
| Woollett et al., 2021 | Zimbabwe Rural  Income level: Lower middle income  Region: Sub-Saharan Africa | To understand pregnant adolescent and young mothers in rural Zimbabwe by describing its risk profile and providing contextually meaningful suggestions for intervention design and implementation | None | Quantitative  Self-administered tablet-administered questionnaires concerning maternal and child health, sexual and reproductive health, psychosocial well-being, and parenting  Purposive sampling | Pregnant and young mothers 14-24 years  n=442 | Psychological well-being: Twenty-four percent of adolescents reported that they often or always feel like they do not have much to be proud of. Sixty-nine percent of children reported that they never or only sometimes count on their friends when they need help. Forty percent reported never having lots of friends, while 16% reported that they are often or always very sad. It is positive to note that 64% of respondents felt that they always or often had at least one adult that loved them and that 63% always or often felt safe at home. Girls reported inadequate social support amidst high caretaking responsibilities and change in relocation for marriage, compromising mental health. Most of the pregnancies were unintended (approximately 60%), which had consequences on attachment and parenting, where roughly 40% of reported difficulties and lack of enjoyment in caring for their babies. |
| Yako, 2007 | Lesotho hospital, health centres, and high school  Income level: Lower middle income  Region: Sub-Saharan Africa | To compare perceived stress in general, stress between a group of unmarried adolescent first-time mothers and a group of married adolescent first-time mothers. | None | Quantitative  Cross-sectional comparative and descriptive  Daily Hassles 53-Item Scale Developed by Kanner, Coyne & Lazarus (1981), Feeling of pregnancy Questionnaire (FOPQ) 78-item Scale Developed by Glazer (1979), and Postpartum Complication Checklist  Convenience Sampling | Adolescent mothers 6 weeks post delivery 15-19 years  n=192 (dyads of 64 unmarried adolescent mothers and their infants, 64 married adolescents and their infants, and 64 high school students) | There were significant differences in perceived stress between both groups of adolescent mothers and the group of never-pregnant adolescents (p <.0001). Never-pregnant adolescents had the lowest levels of perceived stress (p < .0001). Both groups of adolescent mothers had high levels of stress due to pregnancy, and the difference between the two groups was non-significant. |
| Yoosefi Lebni et al., 2020 | Iran, health centers of Kermanshah and Kurdistan  Provinces  Income level: Lower middle income  Region: Middle East & North Africa | To explore the causes and grounds of childbirth fear and the strategies  used by pregnant adolescent women in Iran to overcome such fears. | None | Qualitative  semi-structured interviews  Purposive sampling | Primiparous women, under 18 years  n=15 | Participants reported fear of childbirth with subcategories of fear of child health, fear of childbirth process, fears about inappropriate medical staff performance, fears about hospital environment, and postpartum fears. strategies to reduce childbirth fear with subcategories of choosing appropriate medical centers, increasing information on childbirth, avoiding stressful sources, improving self-care, getting prepared for delivery day in advance and resorting to spirituality. |

*Note: Abbreviations:* AOR = Adjusted Odds Ratio; CIDI = Composite International Diagnostic Interview; COR = Crude Odds Ratio;;EPDS = Edinburgh Postnatal Depression Scale; FI = Food Insecurity; FOPQ = Feeling of Pregnancy Questionnaire; GHQ = General Health Questionnaire; HCW = Health Care Worker; HFIAS = Household Food Insecurity Access Scale; HQ-9 = Health Questionnaire-9; MINI = Mini International Neuropsychiatric Interview; PHQ-9 = Patient Health Questionnaire-9; PND = Postnatal Depression; PPD = Postpartum Depression; PTSD = Post-Traumatic Stress Disorder; SRQ-20 = Self-Reporting Questionnaire-20; USAID = United States Agency for International Development; WHO = World Health Organization.
